# Residue-Level Determination of Small-Molecule–Protein Affinities by Hydrogen–Deuterium Exchange Mass Spectrometry

**DOI:** 10.1021/jasms.6c00020

**Published:** 2026-03-31

**Authors:** De Lin, Luma Godoy Magalhaes, Joel McMillan, Thomas C. Eadsforth, Greg Stewart, Kieran R. Cartmill, Vincent L. G. Postis, Glenn R. Masson

**Affiliations:** † Drug Discovery Unit, Division of Biological Chemistry and Drug Discovery, School of Life Science, 3042 University of Dundee, Dundee DD1 5EH, U.K.; ‡ Division of Cancer Research, School of Medicine, University of Dundee, Dundee DD1 9SY, U.K.

**Keywords:** HDX-MS, HDX-MS/MS, mass spectrometry, peptides and proteins, ligands, ions, hydrogen Isotopes

## Abstract

Hydrogen–Deuterium Exchange Mass Spectrometry (HDX-MS) is an established tool in drug discovery, used to characterize target engagement and conformational dynamics, and frequently used in both biopharmaceutical and small molecule drug discovery. Conventional HDX-MS experiments are performed at saturating ligand concentrations to generate a binding “footprint”, where decreased solvent exchange reflects a local structural stabilization or reduced solvent accessibility upon binding. Here, we present an extended HDX-MS and HDX-MS/MS titration workflow with electron capture dissociation (ECD) fragmentation capable of estimating apparent dissociation constants (*K*_D_^app^) at global, peptide, and single amino acid resolution by fitting uptake-concentration relationships under EX2 exchange and Langmuir binding assumptions. The ability to determine affinity constants in a spatially resolved manner combined with the automation available in HDX-MS sample handling and data analysis enables quantitative mapping of ligand–protein interactions and provides a scalable approach for structure–activity relationship studies in drug discovery.

## Graphical abstract

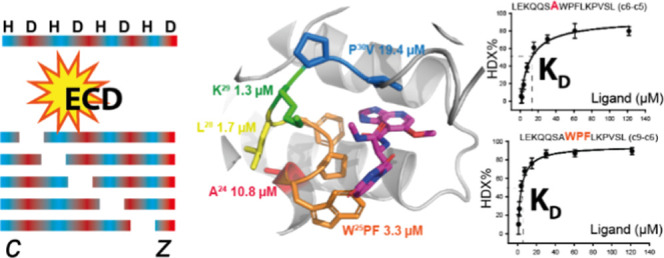

## Introduction

Hydrogen–Deuterium Exchange Mass Spectrometry (HDX-MS) has emerged as a key technique in structure-based drug discovery, enabling a rapid characterization of protein–ligand interactions and conformational dynamics in both biopharmaceutical and small molecule development.^1–8^ Typically, HDX-MS studies follow a “bottom-up” workflow in which the therapeutical target is incubated with a saturating concentration of ligand in the presence of a deuterated buffer for a series of defined time points. Hydrogen–deuterium exchange is then quenched, and the protein chemically denatured. The denatured protein is then proteolytically digested, and the resulting peptides are analyzed by LC–MS to determine the extent of deuterium incorporation across their sequences.^9^ These resulting data provide peptide-level spatial resolution, typically 5–35 amino acids, allowing the identification of regions stabilized or shielded from the solvent upon ligand binding.^10^ Higher resolution, including single-residue resolution, can be achieved computationally, especially when high peptide redundancy and sequence coverage is obtained.^11–13^

To minimize heterogeneous exchange behavior, ligand-binding HDX-MS experiments are commonly conducted at concentrations well above the compound dissociation constant (*K*_D_), producing a fully bound complex to avoid heterogeneous bimodal isotopic envelopes.^2^ This belies the fact that HDX-MS workflows have been previously described which are capable of determining affinity constants, such as PLIMSTEX^14^ and SUPREX,^15^ and a push to make HDX-MS more “quantitative”, *i.e.*, able to abstract meaningful (and crucially comparable) biophysical constants from HDX-MS data.^16^ Furthermore, there has been renewed interest, notably from the Wilson group and co-workers, in HDX-MS/MS (*i.e.*, the fragmentation of a deuterated precursor peptide to produce deuterated product ions), which enables residue-level investigation of deuterated peptides.^17–19^ These experiments build on the comprehensive and groundbreaking work in the groups of Jørgensen and Rand, who used electron capture dissociation (ECD) or electron transfer dissociation (ETD) to achieve fragmentation with minimal hydrogen/deuterium (H/D) scrambling.^20–23^ Although HDX-MS/MS has been applied to ligand binding,^24,25^ its use has remained limited, possibly due to the low efficiency of fragmentation and the stringent controls required to detect signal scrambling. Newer technologies, such as Electron-Activated Dissociation (EAD), have shown improved performance, increasing the accessibility of scramble-free HDX-MS/MS for ligand screening.^17^

Here, we report a compound titration experiment using both HDX-MS and ECD-based HDX-MS/MS that enables the determination of apparent dissociation constants (*K*_D_^app^) with an amino acid spatial resolution. Using the *Plasmodium falciparum* GCN5 bromodomain as a model system, we measured deuterium uptake as a function of compound concentration at the peptide and single-amino acid level to extract quantitative affinity information. Comparison with orthogonal biophysical methods showed a strong agreement between HDX-derived and reference *K*_D_ values. This approach provides a time-efficient, low-sample-consumption, in-solution method of quantifying ligand binding and mapping local conformational changes, enabling detailed characterization of the mode of interaction and binding mechanisms in protein–ligand events.

## Methods

### Materials

Deuterium oxide (99.9%) and formic acid were purchased from Sigma-Aldrich (St Louis, MO, USA). Ultrapure HPLC grade water and acetonitrile were purchased from VWR Chemicals (Radnor, PA, USA). Sodium iodide (2 μg/μL in 50:50 isopropanol/water), 200 pg/nL leucine enkephalin (50:50 acetonitrile/water, 0.1% formic acid), ACQUITY UPLC BEH C18 columns (130 Å, 1.7 μm), Enzymate Protein Pepsin Column (300 Å, 5 μm, 2.1 mm × 30 mm) (Waters Corp), glass sample vials (12 × 32 mm screw neck vial, total recovery, 1 mL volume with cap and preslit PTFE/Silicone Septum), and all fluidics and trap components were obtained from Waters Corp. (Milford, MA, USA).

#### Protein Expression and Purification

The *Plasmodium falciparum* GCN5 bromodomain (residues 1356–1460) (PfGCN5-BRD) was synthesized with an *N*-terminal TEV-cleavable His_6_ tag (twist bioscience) and cloned into a pET29a vector using *Nde*I/*Xho*I restriction sites. The plasmid was transformed into *E. coli* BL21­(DE3) cells and expressed in Terrific Broth supplemented with 50 μg mL^–1^ of kanamycin. Cultures were grown at 37 °C to an OD_600_ of 0.6–0.8, induced with 0.15 mM IPTG, and incubated at 20 °C for 18 h. Cells were harvested and lysed in 20 mM HEPES, 500 mM NaCl, 10 mM imidazole, 1 mM TCEP, 5% (w/v) glycerol, pH 7.5, containing DNaseI and protease inhibitor cocktail (Merck, catalog no. 04693132001), using a continuous-flow cell disruptor (Constant Systems, 30 kpsi).

The His_6_-PfGCN5 bromodomain used for surface plasmon resonance experiments was purified by Ni–NTA affinity chromatography (10–500 mM imidazole gradient elution) followed by size-exclusion chromatography (Superdex 75, Cytiva).

For HDX-MS and HDX-MS/MS studies, the His_6_ tag was removed by TEV protease digestion after initial Ni–NTA purification. The cleaved protein was subjected to reverse Ni–NTA purification and further purified by size-exclusion chromatography (Superdex 75, Cytiva). All protein preparations were concentrated to 10 mg mL^–1^ in 10 mM HEPES, 100 mM NaCl, and 0.5 mM TCEP, pH 7.5, flash-frozen, and stored at −80 °C until use.

#### Surface Plasmon Resonance (SPR)

Surface plasmon resonance (SPR) experiments were performed on a Biacore 8K+ instrument (Cytiva). Flow cell 1 (FC1) served as the reference surface. His_6_PfGCN5-BRD (8 μg/mL) was immobilized on flow cell 2 FC2 of either a NTA-derivatized linear polycarboxylate hydrogel (medium charge density, 1500 M chip Xantec, cat n. SCBS NiHC1500M) or a Biacore Sensor Chip NTA (Cytiva, cat. BR100532) using nickel capture followed by amine coupling (amine coupling kit, Cytiva cat. BR100050). Two channels were used in parallel, yielding typical immobilization levels of approximately 2500 RU (channel 1) and 3000 RU (channel 2) for PfGCN5. The running and dilution buffer contained 25 mM HEPES adjusted to pH 7.5, 150 mM NaCl, and 0.5 mM TCEP.

Compounds were prepared as a six-point, 3-fold serial dilution (top concentration 30 μM, 0.3% DMSO) using an echo acoustic dispenser (Beckman) to pre-stamp a 384-well plate (Greiner, cat. no. 78128) followed by the addition of 100 μL of dilution buffer to the well.

Blank buffer injections (25 mM HEPES pH 7.5, 150 mM NaCl, 0.5 mM TCEP, 0.3% DMSO) were included between compound injections to enable referencing (FC2–FC1 subtraction, followed by compound–blank cycle subtraction). Samples were injected at 30 μL/min over both FC1 and FC2, with a 60 s association phase and a 120 s dissociation phase. Solvent correction was applied using a five-point DMSO calibration series (0.1–0.5%). The control compound (GSK4027 ^26^) was analyzed at the start and end of each run as a six-point, 3-fold dilution (top concentration 10 μM) in both channels. Sensorgrams were processed and fitted using Biacore Insight Evaluation Software v5.0.18.22102. Equilibrium dissociation constants (*K*_D_) were determined from steady-state binding responses.

#### PfGCN5 Crystallization

PfGCN5-BRD (22.6 mg/mL) was mixed with a 2-fold molar excess of DDD02444250 for 30 min on ice. Crystals were grown by sitting-drop vapor diffusion at 18 °C under conditions consisting of 0.2 M sodium chloride, 0.1 M phosphate/citrate pH 4.2, and 20% (w/v) PEG 8000. Prior to flash-cooling in liquid nitrogen, crystals were cryoprotected in a reservoir solution supplemented with 20% (v/v) ethylene glycol.

X-ray diffraction data were collected at 100 K at beamline I04 at the Diamond Light Source with a wavelength of 0.9537 Å. Data were processed using DIALS^27,28^ within the Xia2 pipeline^29^ and data reduction was performed with Aimless.^30^ The structure was solved by molecular replacement with MOLREP^31^ using PDB 5TPX as a search model. Manual building and refinement were performed with Coot^32^ and Refmac5 ^33^ within the CCP4 program suite.^34^ Refinement statistics are included in Supporting Information Table S1. Structural figures were generated in PyMOL.

#### Hydrogen–Deuterium Exchange-Mass Spectrometry

Prior to deuterium exchange experiments, a protein sequence coverage map was generated by analyzing the undeuterated PfGCN5-BRD protein under the following conditions: 60 μL of the 1 μM protein in 20 mM HEPES, pH 7.5, 150 mM NaCl, 0.5 mM TCEP, and 1% (v/v) DMSO was mixed with 50 μL ice-cold quenching buffer (4 M urea, 2% formic acid).

Stocks solution of PfGCN5-BRD 20 μM and PfGCN5-BRD 20 μM preincubated for 5 min at 20 °C with 100 μM compound (DDD02444250) were prepared in 20 mM HEPES pH 7.5, 150 mM NaCl, 0.5 mM TCEP and 1% (v/v) DMSO and maintained at 0.1 °C. These mixtures were equilibrated for 5 min at 20 °C prior to deuterium exchange.

The exchange reaction was performed at 0, 0.5, 5, and 50 min using an automated liquid-handling platform. For each labeling reaction, 3 μL of sample stock was mixed with 57 μL of D_2_O buffer (20 mM HEPES pH 7.5, 150 mM NaCl, 0.5 mM TCEP, and 1% DMSO in 94% D_2_O) at 20 °C, resulting in a final D_2_O content of 89.4% (v/v).

After the labeling, 50 μL of each sample was mixed with 50 μL of 0.1 °C quenching buffer at 0.1 °C for 1 min prior to LCMS data acquisition. The two shortest labeling time points (0.005 and 0.05 min) were prepared manually, as these durations were below the minimum operating cycle of the liquid-handling system. For the 0.005 min exchange time point, the samples were prepared on ice in a 4 °C room with prechilled D_2_O buffer and pipet tips, and the exchange was conducted for 3 s (the ∼20 °C drop in temperature corresponding to a 10-fold reduction in solvent exchange rate (as defined in the Arrhenius Equation^35^)). For the 0.05 min time point, the samples were prepared and incubated with D_2_O buffer for 0.05 min (3 s) at room temperature. Samples were frozen immediately in liquid nitrogen and later stored at −80 °C until further analysis. All deuterium-labeled samples were repeated three times.

For affinity determination, the experiment was conducted by diluting 3 μL of the ligand-free protein stocks (20 μM) with 57 μL of D_2_O buffer containing various compound concentrations of 1, 2, 4, 8, 16, 32, 64, and 128 μM while keeping the total protein concentration constant. The final concentration of PfGCN5-BRD in the labeling reaction was 1 μM. These mixtures were equilibrated for 5 min at 20 °C prior to deuterium exchange. The labeled sample (50 μL of the 60 μL exchange reaction) was mixed with 50 μL of quenching buffer (4 M urea, 2% formic acid) at 0.1 °C for 1 min prior to LCMS data acquisition.

HDX-MS data were acquired using the HDX manager (Waters Corp.) maintained at 0.1 °C and coupled in-line to a SELECT SERIES Cyclic IMS QTOF mass spectrometer (Waters Corp.). Automated liquid handling was performed using a PAL3 robotic tool change system, controlled by Chronos Software (LEAP Technologies). The protein was digested in-line for 2 min at 20 °C using an Enzymate BEH Pepsin 2.1 mm × 30 mm column (Waters Corp.). The generated peptides were desalted in buffer A (0.1% formic acid in water) using an ACQUITY UPLC BEH C18 VanGuard Precolumn (1.7 μm, 5 × 2.1 mm; Waters Corp.) prior to reversed-phase separation on an ACQUITY UPLC C18 column (1.7 μm, 100 × 1 mm; Waters Corp.). Peptides were eluted using a 40 μL/min flow rate from 5% to 85% buffer B (0.1% formic acid in acetonitrile) in buffer A. Data were acquired in positive mode over a *m*/*z* range of 50–1200 with a spray voltage of 2.0 kV with leucine enkephalin (*m*/*z* 556.2766) used as a lockmass internal calibrant during data acquisition.

### Data Processing

Peptide identification and data processing were performed using the ProteinLynx Global Server (PLGS version 3.0.3 Waters Corp.). A reference peptide map was generated from three replicates of nondeuterated samples acquired in HDMSe, V-mode, and 0.4 s scan time, using identical chromatographic and instrumental parameters to those applied for the deuterated samples.

Raw data were processed using HDExpress 3v39,^36^ HDExaminer v3.5.0 (Trajan Scientific and Medical), and Newmarket (unreleased software, Waters Corp.) for deuterium uptake analysis. Reported uptake values were not corrected for back-exchange and, therefore, represent relative deuterium incorporation. All mass spectrometry data have been deposited to the ProteomeXchange Consortium via the PRIDE partner repository with the dataset identifier PXD070051.

#### Derivation of the Local Protein Ligand Dissociation Constant from HDX-MS Data

Equilibrium dissociation constants (*K*_D_^app^) were obtained from steady-state binding response by fitting Δ*D* versus ligand concentration to a Langmuir isotherm (1:1) under EX2 exchange, assuming rapid conformational pre-equilibrium and negligible ligand depletion, *i.e.*, that free ligand [*L*] = total ligand [*L*_0_].

Differences in deuterium uptake as a function of ligand concentration were used to monitor local protein–ligand interactions at peptide- or single-amino acid-level spatial resolution. The percentage of deuterium uptake protection (Δ*D*) was calculated by comparing uptake in the presence and absence of ligand at 5 min of exchange reaction at 20 °C. Data exported from HDExaminer v3.5.0 (Trajan Scientific and Medical) or Newmarket (beta, unreleased software; Waters Corp.) were analyzed using a custom automated Python script (HDX_KD_data_analysis_HDXaminer.py or ECD_KD_all_peptide_final.py respectively). The script parses peptide-level uptake values, aligns data across conditions, calculates differential deuterium uptake (Δ*D*), and generates comparative plots of HDX kinetics. The Python analysis scripts are available at Zenodo (DOI: 10.5281/zenodo.17492311) and maintained on GitHub (https://github.com/Glenn-Masson/De_HDX_KD).

### Targeted ECD

Peptides exhibiting protection upon compound binding in HDX-MS experiments were selected for targeted electron capture dissociation (ECD) fragmentation based on exact mass and retention time and window. ECD cell parameters were optimized to minimize hydrogen/deuterium scrambling using the “P1 peptide” standard (sequence HHHHHHIIKIIK, synthesized by Cambridge Bioscience).^37^ Furthermore, scrambling of the targeted peptide was monitored via measuring for deuterium loss coincident with ammonia ion fragment formation^22^ with measurement of deuterium incorporation on the RS^2+^ and RS^2+^-NH_3_ being conducted using HDExpress 3 v39 ^36^ (See Supporting Information Figure S1). Multiple 0.5 min −1 min Data Dependent Acquisition (DDA) methods (50–2000 *m*/*z* range, 1 s scan time) targeting deuterated precursor masses were used to target peptides for ECD fragmentation (filament set a 3 A). ECD fragment spectra were processed using Newmarket software (Beta, unreleased software, Waters Corp.) and visualized with SigmaPlot v14.5 (Systat Software) and PyMOL v3.0.0 (Schrödinger LLC).

## Results and Discussion

### Dissociation Constants Determined via Peptide-Level HDX-MS Analysis

To demonstrate the capacity of HDX-MS analysis to determine dissociation constants for ligand binding, we used the bromodomain (BRD) of *Plasmodium falciparum* histone acetyltransferase GCN5 (General Control Nonderepressible 5) (PfGCN5) (residues 1356–1460). PfGCN5-BRD is a potential target for the treatment of malaria parasite *Plasmodium falciparum*,^38^ which has a classical bromodomain structure of four alpha helices with two interhelical loops.^39–41^ An in-house drug discovery screen developed the compound DDD02444250 (′4250), which, using surface plasmon resonance (SPR) had a reported *K*_D_ of 4.8 μM (Supporting Information Figure S2).

To determine the site of interaction of ′4250 with PfGCN5-BRD, we conducted HDX-MS using a Trajan LEAP Pal autosampler, with a Waters HDX-Manager upstream of a Waters cIMS instrument fitted with an eMision Electron Capture Dissociation (ECD) cell. We were able to create a peptide map of PfGCN5-BRD consisting of 72 peptides, representing 100% coverage with an 8.7 redundancy score (Figure 1A, Table 1). Differential HDX-MS was performed by incubating 1 μM of PfGCN5-BRD with 20 μM ′4250 prior to deuteration at five different time points (0.005, 0.05, 0.5, 5, and 50 min, with the 0.005 min time point being a 3 s time point conducted on ice) to produce a deuterium uptake difference map between the apo and ligand-bound states (Figure 1B,C,D and Table 1.0). To gain further insight into the interaction between ′4250 with PfGCN5-BRD, we also cocrystallized the protein/ligand complex and solved the structure to 1.80 Å (Figure 1D) using X-ray crystallography (PDB Accession 9TM1) (Supporting Information Figure S3).

**Figure 1:**
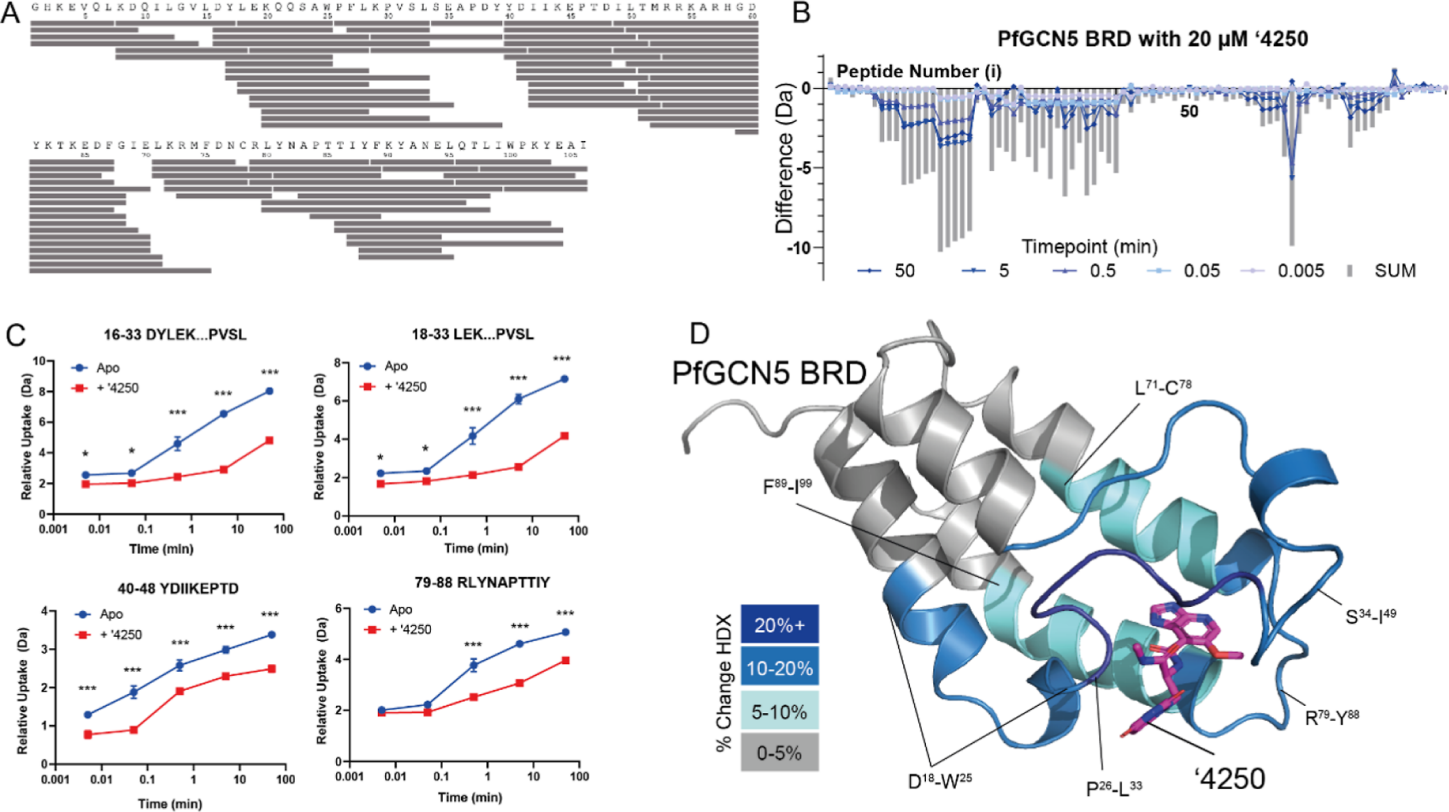
Global shifts in solvent exchange in PfGCN5-BRD on compound binding. (A) Peptide map of PfGCN5-BRD showing coverage of pepsin-produced peptide map. (B) Global HDX profile of PfGCN5-BRD on incubation with 20 μM ′4250. Each time point is shown alongside the sum of the shifts (SUM). (C) Representative peptide uptake plots of four peptides showing changes in ′4250 binding. Each data point is the average with standard deviation shown as error bars (*n* = 3). * = > 5% and >0.5 Da difference and passes a student *t*-test with *p* < 0.05, **p < 0.01, and ***p < 0.001. (D) Shifts in HDX mapped onto the PfGCN5-BRD:′4250 cocrystal structure, solved to 1.8 Å resolution. PDB: 9TM1.

**Table 1:** HDX-Statistics Table for ′4250 Binding to PfGCN5-BRD^a^

	apo	'4250
# of peptides	72	72
coverage	100%	100%
average peptide length	12.8 aa	
redundancy	8.7	
time points (min)	0.005 *, 0.05, 0.5**, 5**, 50 **	
replicates	ND 2, deuterated 3	
replicability	0.89%	
significance threshold	>5%/0.5 Da/passes *t* test	

a * = 0.005 min time point conducted as a 3 second timepoint on ice. ** = conducted using the LEAP liquid handling robot.

Next, we sought to determine whether the apparent dissociation constants (*K*_D_^app^) of compound ′4250 for PfGCN5-BRD could be quantified by HDX-MS titration. A 128 μM ′4250 solution was prepared in a deuterated buffer, which was then serially diluted 1:1 eight times in deuterated buffer to create a compound titration series in deuterated buffer. This compound series in deuterated buffer was then incubated with 1 μM of PfGCN5-BRD for a single 5 min time point (Figure 2) (the final top concentration of ′4250, after the addition of PfGCN5-BRD solution, was 120 μM) prior to quenching and mass analysis as previously described. This labeling period was chosen to maximize the dynamic range of measurable protection, with the greatest number of peptides exhibiting the largest differences in deuterium uptake upon compound binding, whereas differences in H/D exchange kinetics converged at shorter and longer labeling intervals. In total, 40 of 72 (56%) of all peptides had a statistically significant difference at the 5 min time point on ′4250 binding, and for 27 of those peptides, the 5 min time point had the largest Δ*D*. Only one peptide (^71^LRKMFGNC^78^) had a significant uptake difference on compound binding at a time point (50 min) where there was not also a significant Δ*D* at 5 min, but redundant neighboring peptides still provided insight into this region for *K*_D_^app^ studies.

**Figure 2:**
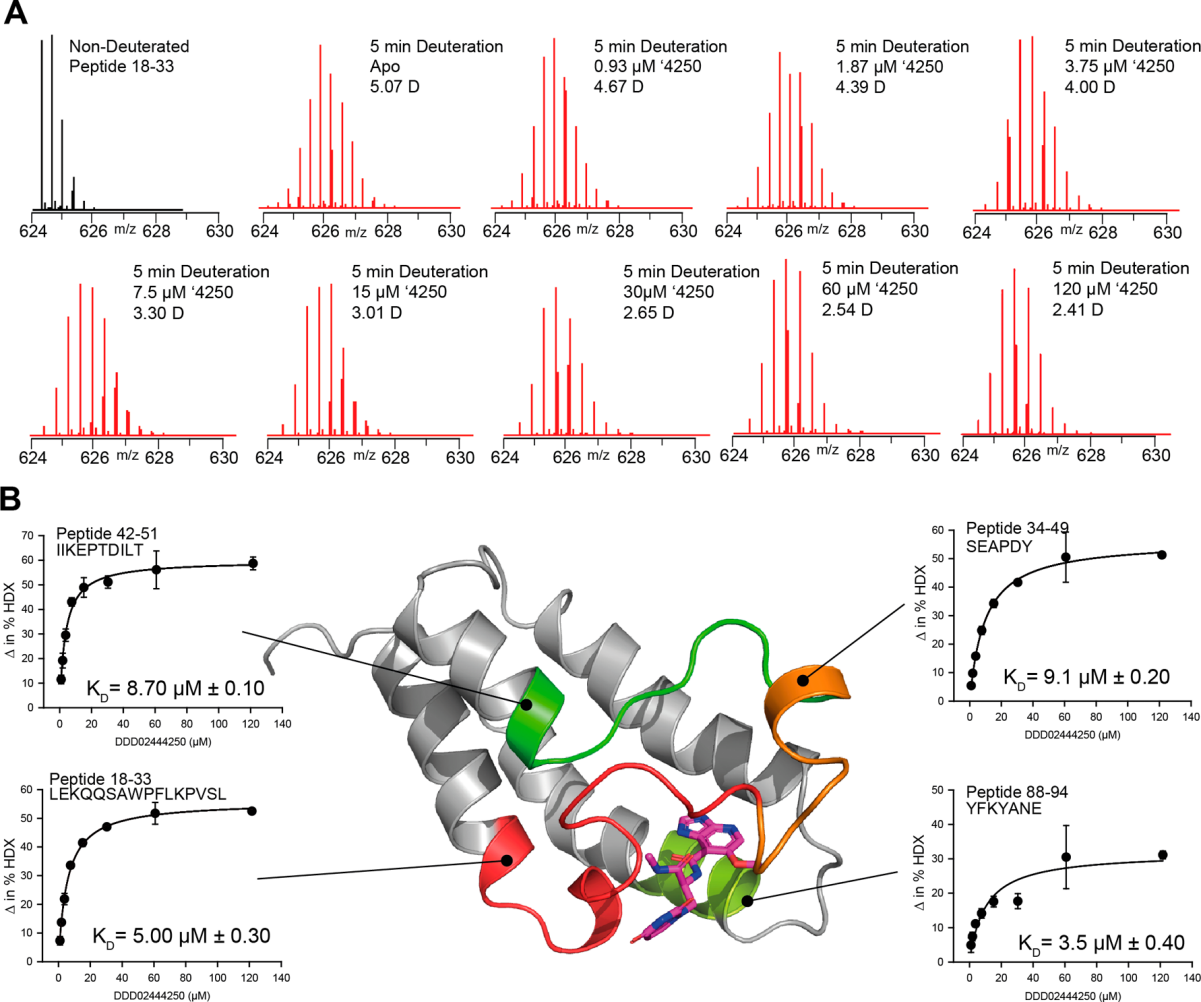
Peptide-Level HDX-MS to derive spatial *K*_D_^app^. (A) Representative spectra of peptide 18–33 at the 5 min deuteration time point across 8 concentrations of ′4250 (B) spatial mapping of the *K*_D_^app^ values onto 4 representative peptides from the data set on the crystal structure PDB 9TM1. In total, the *K*_D_^app^ values for 40 peptides could be determined (see Table 2). Each data point in the uptake curves is the average of three independent exchange reactions with standard deviation shown (*n* = 3).

Δ*D* values representing the change in deuterium uptake between apo and ligand-bound PfGCN5-BRD were obtained at the 5 min labeling time point for each compound concentration in the titration series. Apparent dissociation constant (*K*_D_^app^) values were determined by fitting plots of Δ*D* versus ligand concentration to a single-site Langmuir equation

$$\Delta D=\frac{{\Delta D}_{\max}\left[L\right]}{K_{D}+\left[L\right]}$$

where Δ*D*_max_ is the fitted maximal deuterium uptake, [*L*] is the ligand concentration, and *K*_D_ is the fitted local equilibrium dissociation constant. We assumed that free ligand concentration ([*L*]) was equal to that of total ligand ([*L*_0_]), i.e., there was negligible ligand depletion on protein binding. Nonlinear regression was performed using a custom python script (HDX KD data analysis HDXaminer.py), and the coefficient of variation (CV) of Δ*D*_max_ was calculated using the standard deviation from curve fitting to assess fitting precision. A selection criterion was applied such that a *K*_D_ value was accepted only if Δ*D*_max_ was at least 20% and its CV was below 20%.

The resulting titration data yielded characteristic binding curves, showing a plateau in deuterium exchange protection as a function of compound concentration. From these fits, we obtained an apparent dissociation constant (*K*_D_^app^) from the midpoint of each curve. A total of 40 peptides produced measurable *K*_D_^app^ values for compound ′4250, many representing overlapping sequence regions of PfGCN5-BRD, providing high internal consistency (Table 2). For example, three peptides with a high degree of redundancy (residues 87–94, 88–93, and 89–95) all exhibited *K*_D_^app^ values of 2.3–3.5 μM, while four peptides covering residues 16–33 (peptides 16–25, 16–33, 17–33, and 17–25) all exhibited a *K*_D_^app^ values of 4.3–4.9 μM. The mean (*K*_D_^app^) was 5.5 ± 4.1 μM, in close agreement with the reference value obtained by SPR (4.8 μM Cl = 4.0–5.8 95%). Individual *K*_D_^app^ peptide-derived values ranged from 2.3 ± 0.4 μM (residues 72–80) to 30.5 ± 6.1 μM (residues 42–49). These results demonstrate strong concordance between peptide-level HDX-MS-derived affinities and orthogonal biophysical measurements, supporting the reliability of this approach for quantifying ligand binding in solution. A related question is whether the maximum protection factor obtained from deuterium buildup curves at full ligand saturation correlates with *K*_D_^app^ values derived from titration experiments; preliminary data suggest a qualitatively positive relationship at the peptide level, but rigorous testing across a broader affinity range is required before this can be used as a predictive tool.

**Table 2:** Dissociation Constants for Peptides Exhibiting a Shift on ′4250 Incubation^a^

ligand	sequence	GCN5 residue range	*K*_D_^App^ (μM)
DDD02444250	DYLEKQQSAWPFL	16–28	3.80 ± 0.20
	DYLEKQQSAW	16–25	4.30 ± 0.30
	DYLEKQQSAWPFLKPVSL	16–33	4.50 ± 0.20
	YLEKQQSAWPFLKPVSL	17–33	4.90 ± 0.20
	YLEKQQSAW	17–25	4.40 ± 0.30
	YLEKQQSAWPFL	17–28	3.60 ± 0.20
	LEKQQSAWPFLKPVS	18–32	5.20 ± 0.20
	LEKQQSAWPFL	18–28	3.70 ± 0.20
	LEKQQSAW	18–25	4.40 ± 0.30
	LEKQQSAWPFLKPVSL	18–33	5.00 ± 0.30
	EKQQSAWPFLKPVSL	19–33	5.20 ± 0.30
	EKQQSAWPFL	19–28	3.40 ± 0.10
	KQQSAWPFLKPVSL	20–33	5.90 ± 0.40
	KQQSAWPFL	20–28	3.50 ± 0.20
	PFLKPVSL	26–33	6.20 ± 0.20
	SEAPDY	34–39	9.10 ± 0.20
	YDIIKEPTDI	40–49	5.00 ± 0.60
	YDIIKEPTDILT	40–51	4.40 ± 0.20
	YDIIKEPTDIL	40–50	4.70 ± 0.30
	YDIIKEPTD	40–48	7.00 ± 1.20
	DIIKEPTDILT	41–51	5.00 ± 0.30
	DIIKEPTDIL	41–50	6.50 ± 0.80
	IIKEPTDILT	42–51	8.70 ± 1.10
	IIKEPTDIL	42–50	10.0 ± 1.7
	IIKEPTDILTM	42–52	4.70 ± 0.30
	IIKEPTDI	42–49	30.5 ± 6.1
	IKEPTDIL	43–50	4.70 ± 0.70
	EPTDILTMR	45–53	6.30 ± 0.50
	PTDILTMRRKA	46–56	3.40 ± 0.40
	LKRMFDNCRL	71–80	2.40 ± 0.40
	KRMFDNCRL	72–80	2.30 ± 0.40
	KRMFDNC	72–78	3.00 ± 1.20
	RMFDNCRL	73–80	2.50 ± 0.70
	RLYNAPTTIY	79–88	6.10 ± 0.20
	YNAPTTIYF	81–89	7.10 ± 0.40
	IYFKYANE	87–94	3.30 ± 0.40
	YFKYANE	88–94	3.50 ± 0.40
	YFKYANEL	88–95	2.30 ± 0.40
	FKYANEL	89–95	2.90 ± 0.70
	FKYANELQT	89–97	2.40 ± 0.60

a Each *K*_D_ value is derived from three independent exchange reactions (*n* = 3).

One aspect of the HDX-MS approach of determining spatial affinity constants is that most commonly a “global” *K*_D_ is determined using other orthogonal methods, such as SPR. By averaging our peptides for this data set, we have attempted to reduce the spatial component of our *K*_D_^app^ to a single value to allow for comparison with these methods. However, there are two issues with this approach: (i) the issue of spatial oversampling due to the redundancy of the peptides (*i.e.*, overlapping peptides count “twice”), and (ii) how to address that many peptides exhibit no Δ*D* and thus their *K*_D_^app^ approximates infinity. In this example of ′4250 binding to PfGCN5-BRD, we took both a maximal (*i.e.*, all peptides) and a minimal approach (where the smallest number of peptides, while maintaining high levels of coverage, in this case 7 peptides (16–33, 26–33, 34–39, 40–51, 71–80, 81–89, and 89–95) to determine the “Global” *K*_D_^app^ and found little difference—the “minimal dataset” *K*_D_^app^ was 5.1 μM (*cf.*, 5.5 μM for maximal).

For peptides showing no measurable decrease in deuterium uptake upon compound binding, no *K*_D_^app^ could be determined (32 peptides, 45% of the data set). The titration experiment was repeated at the 0.5 and 50 min labeling times to evaluate the effect of exchange duration on the calculated *K*_D_^app^. At 50 min, some deuteration curves converged (*e.g.*, 16–25, 34–39, and 42–48) preventing *K*_D_^app^ estimation. Similarly, at 0.5 min, in some peptides, the deuteration rates had not diverged sufficiently between bound and unbound states to provide reliable fitting of the binding curve (71–80, 71–78, 72–80, 88–95). These observations highlight the importance of selecting an optimal labeling time that maximizes the dynamic range of measurable protection; future implementation of an automated time-point optimization tool would facilitate rapid identification of conditions most suitable for *K*_D_^app^ determination across all peptides.

### Dissociation Constants Determined via Peptide-Level HDX-MS/MS Analysis

We next sought to determine whether ECD could be used under minimized H/D-scrambling conditions to determine apparent dissociation constants (*K*_D_^app^) at single-amino-acid resolution. The source and ECD cell parameters were optimized for minimal deuterium scrambling, initially using the P1 peptide HDX scrambling monitor to tune as previously described^21,24,37,42^ with further refinement being conducted using the loss of ammonia method as described by Rand et al.^22^ (Supporting Information Figure S1). Targeted ECD fragmentation was then applied to deuterated precursor ions of the PfGCN5-BRD peptide containing residues 18 to 33. This peptide, detected predominantly in +3 charge state and with a relative abundance exceeding 10^5^ counts, produced an almost complete *c* ion series including *c*3-*c*7 and *c*9-*c*11 and *z* ion fragments *z*9-*z*13, providing a near-complete single-residue coverage (Figure 3).

**Figure 3:**
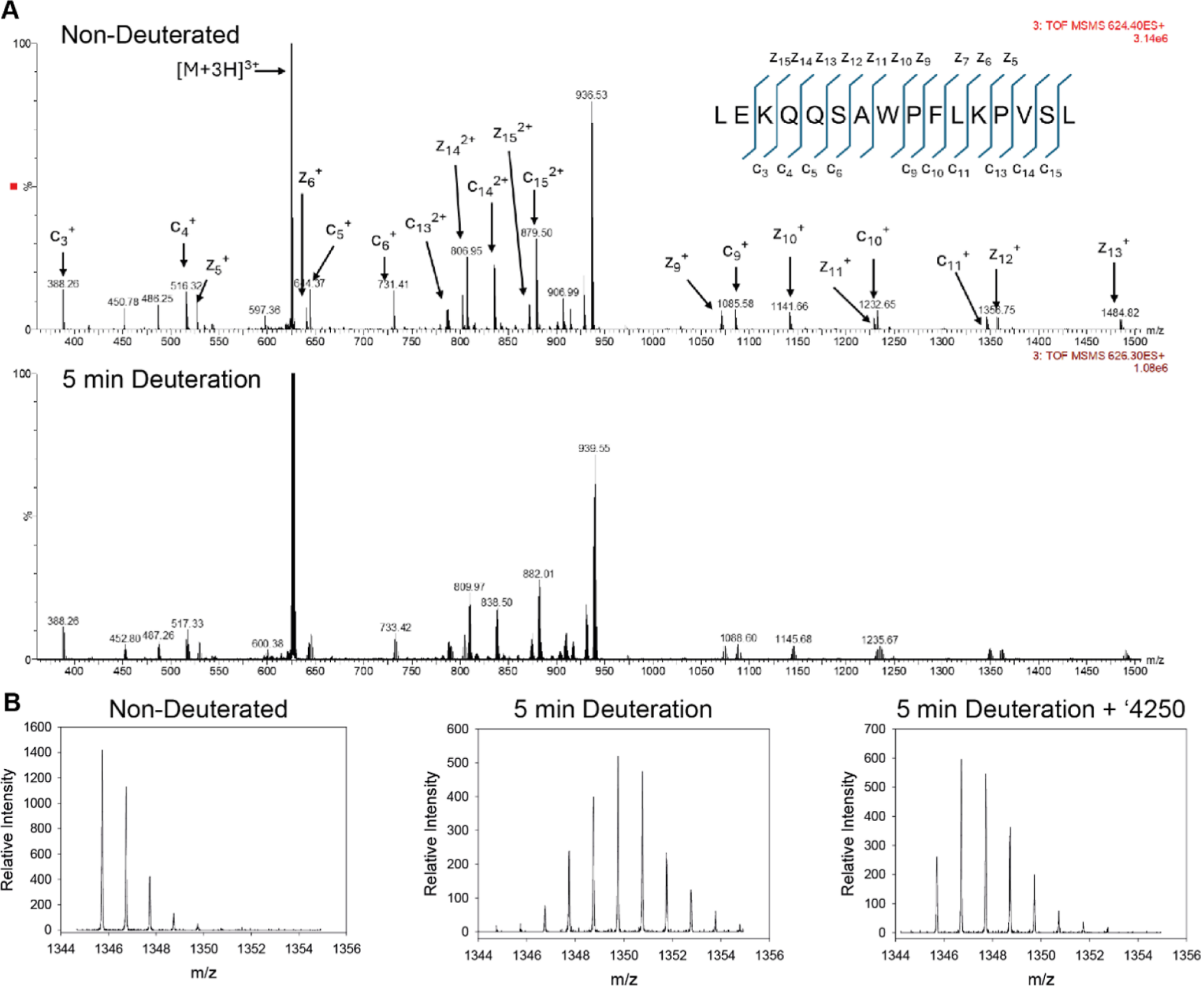
Representative ECD MS/MS spectra of the [M + 3H]^3+^ precursor ion of the peptide LEKQQSAWPFLKPVSL acquired under nondeuterated and deuterated conditions. (A) ECD spectrum of the nondeuterated peptide at 0 s exchange (precursor *m*/*z* 624.40, 3+) and ECD spectrum following 5 min deuterium labeling (precursor *m*/*z* 626.30, 3+). Spectra are displayed at the indicated relative intensity scales. Predominantly *c*- and *z*-type ions consistent with nonergodic ECD cleavage were observed. (B) High-resolution isotopic distributions of a representative ECD fragment ion (c_11_) of the peptide LEKQQSAWPFLKPVSL under nondeuterated and deuterated conditions with and without 120 μM compound ′4250.

Compound titration of ′4250 across the same eight-point concentration curve as used for peptide-level HDX-MS enabled the determination of residue-level *K*_D_^app^ values using HDX-MS/MS for the interacting region A24-V31 (Figure 4). The apparent affinities ranged from 19.4 μM for P30 V to 1.3 μM for K29. Notably, this stretch of amino acids contains two proline residues, P30 and P25, which are “invisible” to HDX-MS/MS analysis due to the absence of backbone amide hydrogens. The HDX-MS/MS analysis therefore not only localized the ligand binding interface between PfGCN5-BRD and ′4250 but also provided quantitative, residue-specific affinity information.

**Figure 4:**
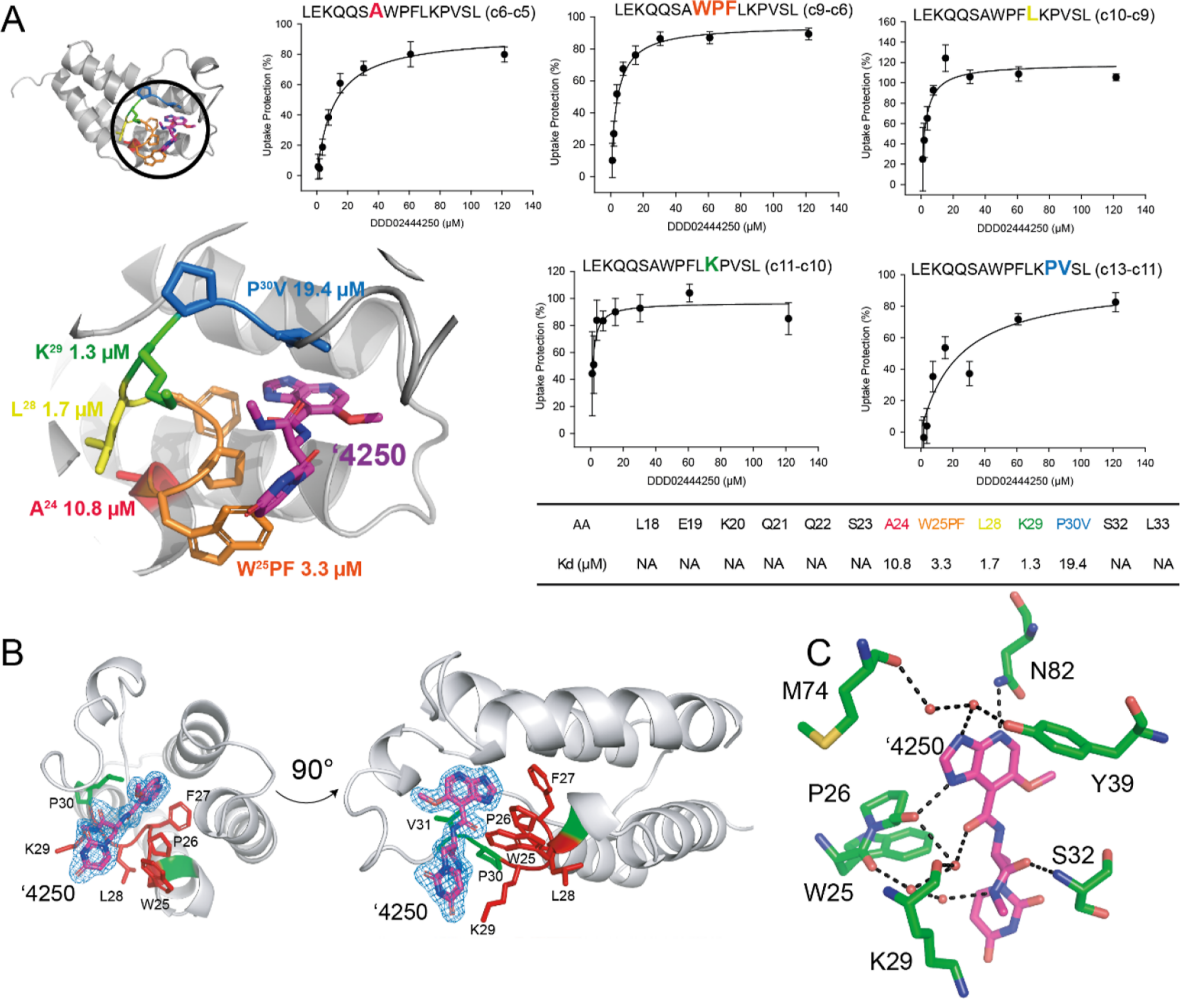
Single amino-acid HDX-MS to derive spatial *K*_D_^app^ at a single residue resolution compared to crystallography data. (A) HDX-MS/MS was conducted using targeted ECD on a single peptide while titrating the ′4250 compound. Consecutive fragments are used to determine individual exchange rates, from which % deuteration can be derived. Each data point is averaged with standard deviation, from *n* = 3, with each exchange reaction being conducted independently. (B) Detail of ′4250 electron density binding to PfGCN5-BRD with amino acids pertinent to subsequent HDX-MS/MS analysis colored red/green. (C) Map of potential polar contacts between ′4250 and PfGCN5-BRD as identified via crystallography. Water molecules shown as red spheres.

Comparing the crystal structure of ′4250 with PfGCN5-BRD to the HDX-MS/MS there is a high degree of agreement (Figure 4B,C). There are several contacts between ′4250 and the amino acids identified in the HDX-MS/MS, although residues M74, N82, and Y39, which interact with the purine derivative part of ′4250 are not present in the HDX-MS/MS data set, we can observe that W25/K29 and S32 are present (as is the “invisible” P26). The site identified as the “tightest” interaction, K29, which had a *K*_D_^app^ of 1.3 μM, has a backbone hydrogen bond to a coordinated water molecule. Interestingly, there is a network of three water molecules which mediate the interaction between P26, W25, K29, and ′4250. It is possible that the presence of ′4250 slows the solvent exchange rate by stabilizing these water molecules at the site of interaction, thus slowing the solvent exchange rate. S32 was not observed to have an interaction with ′4250 via HDX-MS/MS, but the crystal structure suggests that S32’s amide group has a hydrogen bond with ′4250. The fact that this was not observed via HDX-MS/MS is interesting, as it would be expected that, given HDX measures the rate of solvent exchange on the peptide bond amide, one would expect that this residue would experience a high degree of solvent exchange rate protection on compound binding. In the “apo” structure of PfGCN5-BRD (PDB:4QNS),^43^ there is no hydrogen-bonded water associated with this amide group, perhaps meaning there is no exchange event here to disrupt, or potentially, the rate of solvent exchange is slower than our 5 min time point, meaning that this interaction was outside the kinetic window of the HDX-MS/MS experiment.

## Conclusion

Recent advances in peptide fragmentation technologies for HDX-MS/MS^2,17,18,44^ have renewed interest in achieving residue-level localization of deuterium uptake. Here, we demonstrate that HDX-MS/MS using ECD can not only localize ligand binding sites but, through compound titration at selected exchange time points, also be used to derive apparent dissociation constants (*K*_D_^app^). This represents a novel and generally applicable in-solution approach to quantifying ligand affinity without the need for protein tagging or immobilization.

HDX-MS has been used before to derive affinity constants, with studies such as PLIMSTEX^14^ being capable of determining protein/ligand affinity and SUPREX^15^ focusing on protein/DNA affinity. However, both of these approaches essentially measured the entire protein’s change in deuteration uptake on ligand binding, meaning that while a high-quality, in-solution affinity constant using HDX-MS could be obtained, the spatial dimension afforded by middle-down HDX-MS had been lost. Using a HDX-MS (as opposed to HDX-MS/MS) workflow, we were able to assess the local *K*_D_^app^ for ligand binding at a peptide level resolution. This workflow is simple, can be conducted using commercial HDX-MS systems, and offers a rapid and automatable means of determining in solution *K*_D_^app^.

An interesting aspect of applying a titration-based HDX-MS approach is that it produces a distribution of apparent affinity constants across the protein sequence rather than a single value. This may seem paradoxical as ligand binding is a classical 1:1 equilibrium between a single bound and a single unbound state;^45^ thus all residues in the protein should in principle report the same *K*_D_. The apparent heterogeneity in *K*_D_^app^ observed here, therefore, requires interpretation.

Under EX2 conditions, the deuterium uptake at any given amide hydrogen reflects a time-averaged occupancy-weighted average of the local conformational flexibility in the bound and unbound states. For residues directly contacting the ligand backbone—where binding directly suppresses amide hydrogen exchange through steric occlusion or hydrogen bonding—the Δ*D* signal will closely track ligand occupancy, and the fitted *K*_D_^app^ will most faithfully reflect the thermodynamic binding constant. For residues that are protected indirectly, through ligand-induced conformational stabilization propagated across the binding interface, the value of Δ*D*_max_ at saturation will be smaller (because the per-residue contribution of stabilization is lower), and the associated *K*_D_^app^ may deviate from the global value as a function of how cooperatively that stabilization is coupled to ligand occupancy. Furthermore, residues that are exchanging on time scales faster or slower than our fixed exchange time point may be systematically under- or over-represented in their apparent sensitivity to the binding event. The fact that the mean *K*_D_^app^ across all peptides approximates the SPR-derived *K*_D_ is encouraging, but this agreement rests on a single compound–protein pair and should be tested systematically. Finally, a degree of cooperativity is expected to operate within these measurements: once the ligand has occupied the binding pocket, neighboring residues that have little direct contact with the compound are nonetheless more likely to be conformationally restrained, and this indirect effect will be reflected in their *K*_D_^app^ values. Further experimentation across a broader array of protein targets and binding modalities, including allosteric modalities in nonequilibrium environments^46^ is required to develop a predictive framework for interpreting the spatial *K*_D_ distribution in terms of the underlying interaction chemistry.

A key advantage of HDX-MS/MS is its ability to resolve affinity information at single-amino-acid spatial resolution, providing insight into local modes of interaction that complement traditional Structure––Activity Relationship (SAR) studies. The combination of HDX-MS-derived spatial interaction mapping with conventional SAR analysis can accelerate hit validation and early lead optimization by linking the compound structure to localized protein response. The lead optimization stage of drug discovery often requires a medium-throughput structural biology approach to determine how adjustments in lead compounds alter the target engagement. HDX-MS/MS is well suited to facilitate a semiautomated in-solution approach to screen these relatively small (10–30) compound series where both *K*_D_^app^ measurements on a peptide and amino acid level can inform likely compound engagement profiles, especially if coupled to HDX-MS/MS restraint-guided drug docking simulations.

The peptide-level *K*_D_^app^ determination approach described in this study is compatible with standard HDX-MS instrumentation and can be readily adapted to other protein–ligand systems, particularly where in-solution binding measurements are advantageous. The affinity measurement can also be conducted without the use of protein affinity tags (such as His/Strep-tags) or the need for fluorescent moieties, which may introduce artifacts into the experiment. Future developments in automated ECD data acquisition and analysis are expected to further improve throughput and robustness, expanding the applicability of this approach in drug discovery.

## Supplementary Material



## Data Availability

All the mass spectrometry data and data analysis files have been deposited to the ProteomeXchange Consortium via the PRIDE partner repository with the data set identifier PXD070051. X-ray Crystallography Structure deposited to the Protein Databank PDB: 9TM1.

## Notes

Monitoring of scrambling using ammonia loss for HDX-MS/MS of peptide LEKQQSAWPFLKPVSL; SPR determination of dissociation constant of ′4250 for PfGCN5-BRD; X-ray crystallography asymmetric unit of PfGCN5-BRD showing three chains (A/B/C) all bound to ′4250; X-ray crystallography data collection and refinement statistics; and deuteration uptake graphs of PfGCN5-BRD with 120 μM ‘4250 (PDF)
