# Supplementary material for: Residue-Level Determination of Small-Molecule–Protein Affinities by Hydrogen–Deuterium Exchange Mass Spectrometry
Source: J Am Soc Mass Spectrom. 2026 Mar 31;37(6):1391–401. doi: 10.1021/jasms.6c00020 (PMC13237772; doi:10.1021/jasms.6c00020)
Supplement: Supplementary file 1 [file js6c00020_si_001.pdf]

SUPPORTING INFORMATION

TITLE: Residue-Level Determination of Small Molecule–Protein Affinities by Hydrogen–Deuterium Exchange Mass Spectrometry

AUTHORS: De Lin[1], Luma Godoy Magalhaes[1], Joel McMillan[1], Thomas C. Eadsforth[1], Greg Stewart[1], Kieran R. Cartmill[1], Vincent L. G. Postis[1*], Glenn R. Masson[2*]

[1] Drug Discovery Unit, Division of Biological Chemistry and Drug Discovery, School of Life Science, University of Dundee, Dundee, DD1 5EH, UK.

[2] Division of Cancer Research, School of Medicine, University of Dundee, Dundee, DD1 9SY, UK

KEYWORDS HDX-MS, HDX-MS/MS, Mass Spectrometry, Peptides and Proteins, Ligands, Ions, Hydrogen Isotopes

vpostis001@dundee.ac.uk

gmasson001@dundee.ac.uk

**Supplemental Tables and Figures**	**Page**
**Figure S1:** *Monitoring of Scrambling using Ammonia loss for HDX-MS/MS of peptide LEKQQSAWPFLKPVSL*.	S-3
**Figure S2:** *Surface Plasmon Resonance (SPR) determination of dissociation constant of '4250 for PfGCN5-BRD.*	S-4
**Figure S3:** *X-ray crystallography asymmetric Unit of PfGCN5-BRD showing three chains (A/B/C) all bound to '4250*	S-5
**Table S1***: X-ray crystallography data collection and refinement statistics*	S-6
**Figure S4:** *Deuteration uptake graphs of PfGCN5-BRD with 120 μM '4250.*	S-7-14

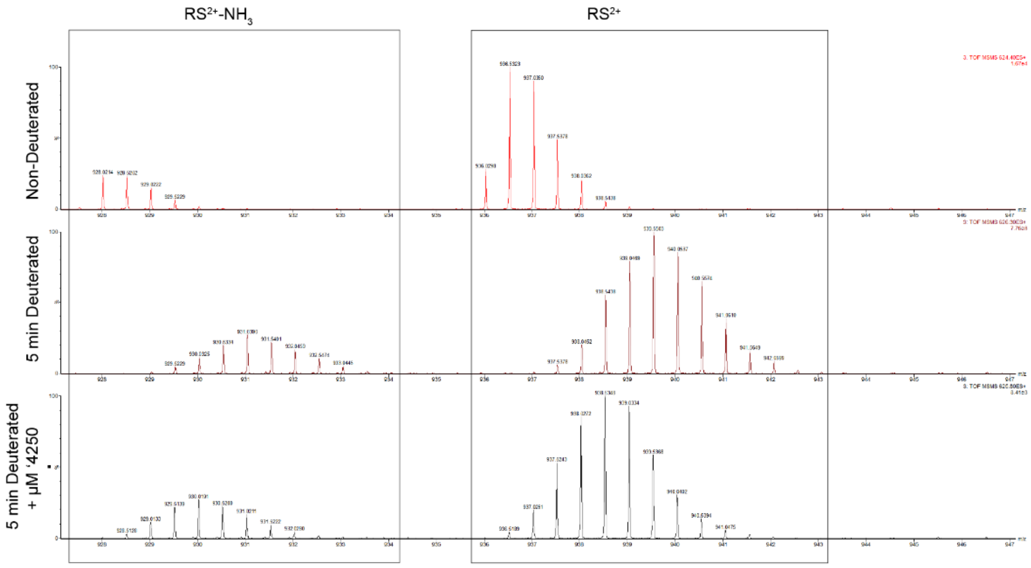

Peptide Sequence	*z*	*no. of sites*[a]	D_{pept} (Da)	$D_{pept\text{-}NH3}$ (Da)	exptl D_{loss} ± error[b]	Calc D_{loss} (Da)[c]
LEKQQSAWPFLKPVSL	3	33	5.1	5.1	0.05 ± 0.10	0.46
LEKQQSAWPFLKPVSL (+ 100 µM '4250)	3	33	3.4	3.3	0.07 ± 0.11	0.30

Supplementary Figure 1: *Monitoring of Scrambling using Ammonia loss for HDX-MS/MS of peptide LEKQQSAWPFLKPVSL*. Representative MS/MS Spectra of Non-deuterated, 5 min deuterated and 5 min deuterated with '4250 compound. The same instrument settings were maintained between experiments. The precursor change state (+3) was reduced with a single electron (RS^{2+}), alongside an associated RS^{2+}-NH_3 counterpart spectra. Deuteration levels of these spectra were determined; if scrambling were to occur, there would be loss of deuterium on the departing NH_3 group [22] . [a]Number of labile hydrogens on the precursor gaseous ion. [b] The average difference between D_{pept} and $D_{pept\text{-}NH3}$ as calculated between the three independent exchange repeats. [c] The theoretical loss of deuterium that a deammoniated peptide would produce given a case of 100% scrambling. This can be calculated by (D_{pept} / (no of sites))*3.

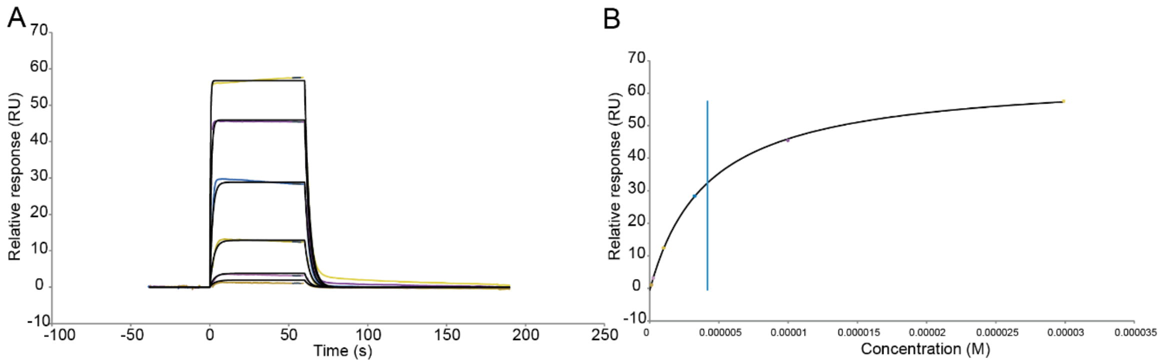

Supplementary Figure 2: *Surface Plasmon Resonance (SPR) determination of dissociation constant of '4250 for PfGCN5-BRD.* (**A**) A 6-point concentration curve showing 1:1 binding of '4250 to immobilized PfGCN5-BRD. (**B**) Dissociation Constant determination of '4250 based on maximal steady state response obtained in (A). Blue line represents mid-point of the curve and associated dissociation constant. Data representative of three independent repeats.

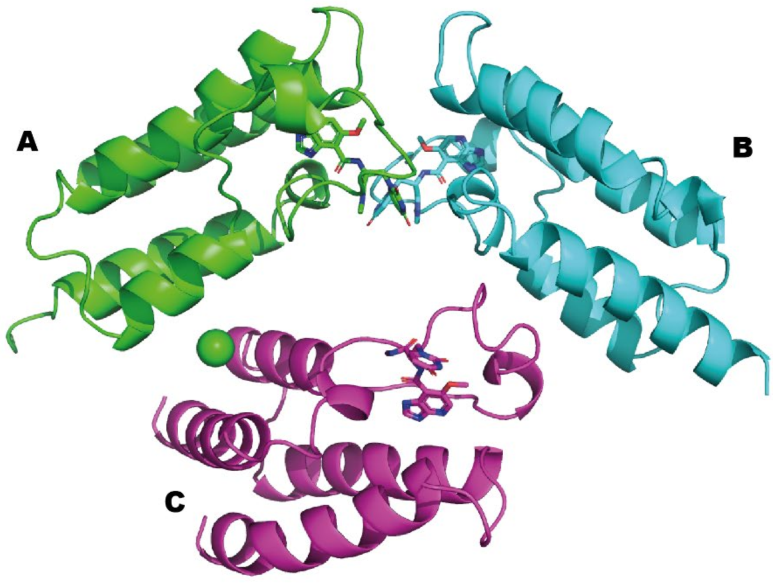

Supplementary Figure 3*: X-ray crystallography asymmetric Unit of PfGCN5-BRD showing three chains (A/B/C) all bound to ‘4250*. Chain A was used for subsequent analysis and image generation in the manuscript.

Supplementary Table 1: X-ray crystallography data collection and refinement statistics

	DDD02444250
**Data collection**	
Source	DLS I04
Wavelength (Å)	0.9537
Space group	$P2_12_12$
Cell dimensions	
a, b, c (Å)	58.08, 165.49, 35.97
α, β, γ (°)	90, 90, 90
Resolution range (Å)	55.16-1.8 (1.84-1.80)*
Total no. of reflections	431491 (22764)
Unique reflections	33196 (1900)
R_{merge}	0.158 (2.120)
R_{pim}	0.064 (0.920)
$I / \sigma I$	9.5 (1.3)
CC1/2	0.998 (0.605)
Completeness (%)	100.0 (99.9)
Redundancy	13.0 (12.0)
**Refinement**	
Resolution (Å)	1.80
R_{work} / R_{free}	19.83 / 24.58
No. atoms	
Protein (A/B/C)	1770/1773/1701
Ligand (A/B/C)	42/42/42
Water/chloride	229/1
B-factors	
Protein (A/B/C)	32.0/32.7/38.4
Ligand (A/B/C)	30.3/28.6/45.5
Water/chloride	35.5/33.3
R.m.s. deviations	
Bond lengths (Å)	0.0076
Bond angles (°)	1.6435
PDB code	

*Values in parentheses are for highest-resolution shell.

GCN5_PLASMODIUM FALCIPARUM 1-7: GHKEVQL (#1)

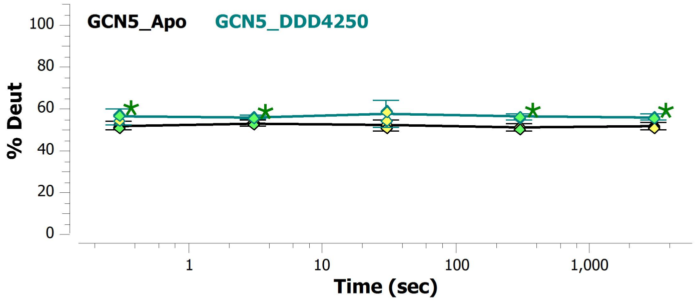

GCN5_PLASMODIUM FALCIPARUM 1-9: GHKEVQLKD (#2)

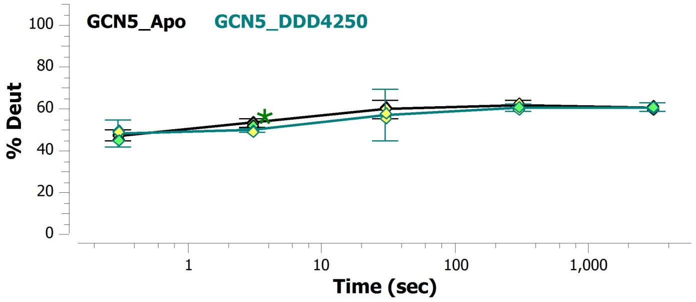

GCN5_PLASMODIUM FALCIPARUM 1-12: GHKEVQLKDQIL (#3)

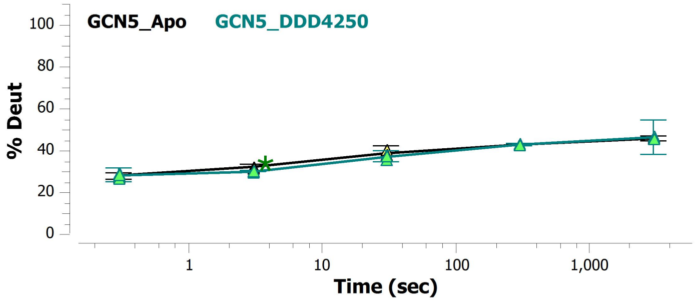

GCN5_PLASMODIUM FALCIPARUM 1-14: GHKEVQLKDQILGV (#4)

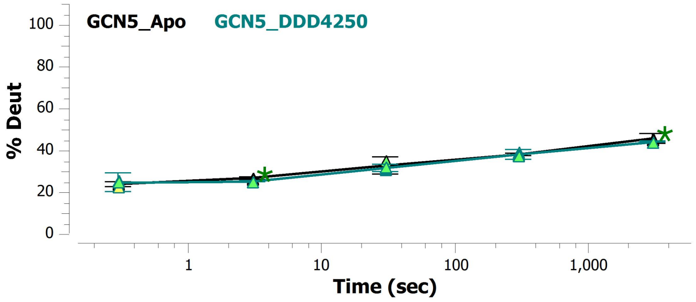

GCN5_PLASMODIUM FALCIPARUM 8-17: KDQILGVLDY (#5)

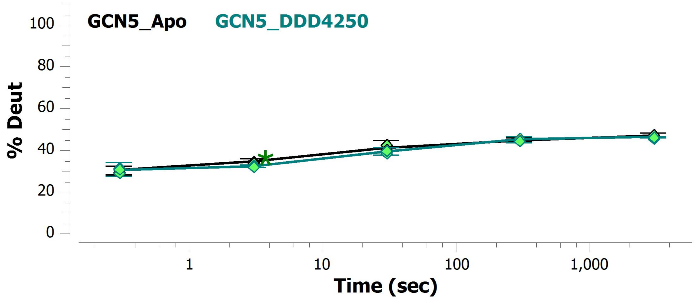

GCN5_PLASMODIUM FALCIPARUM 8-18: KDQILGVLDYL (#6)

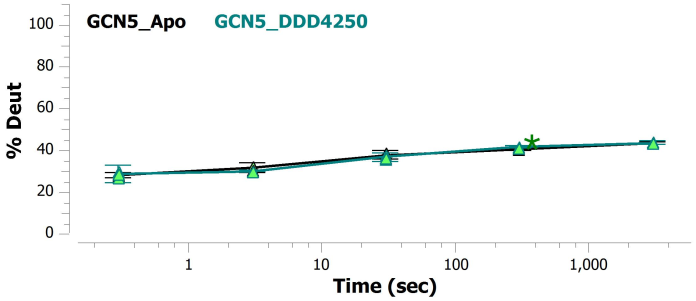

GCN5_PLASMODIUM FALCIPARUM 16-25: DYLEKQQSAW (#7)

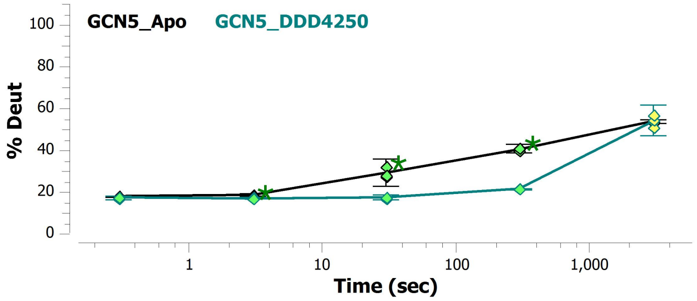

GCN5_PLASMODIUM FALCIPARUM 16-28: DYLEKQQSAWPFL (#8)

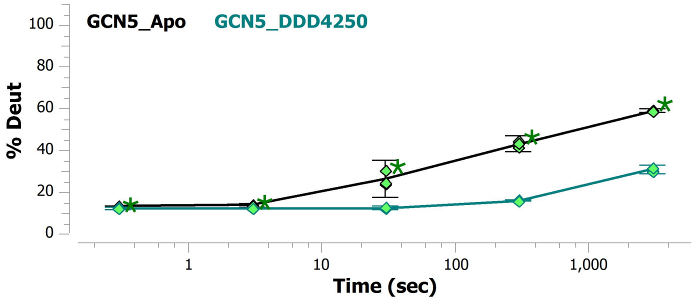

GCN5_PLASMODIUM FALCIPARUM 16-33: DYLEKQQSAWPFLKPVSL (#9)

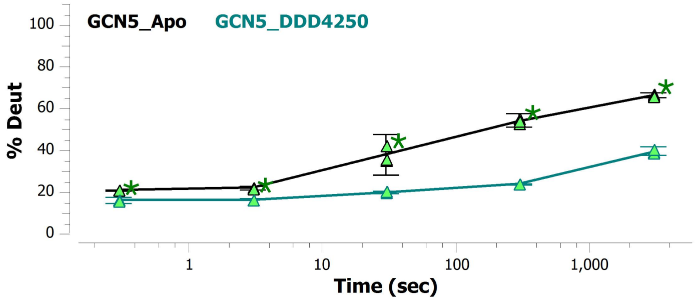

GCN5_PLASMODIUM FALCIPARUM 17-25: YLEKQQSAW (#10)

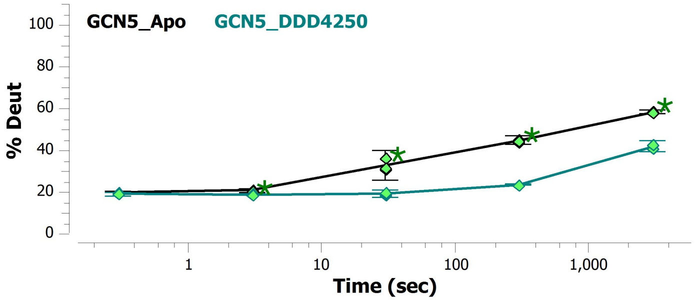

GCN5_PLASMODIUM FALCIPARUM 17-28: YLEKQQSAWPFL (#11)

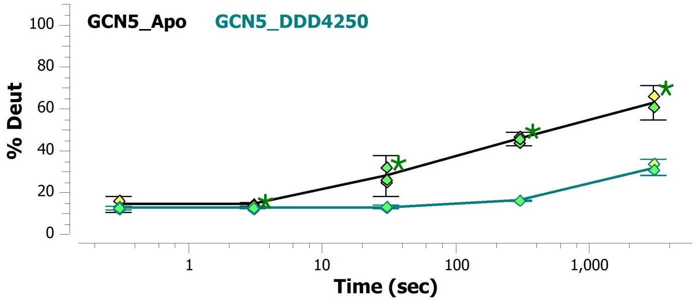

GCN5_PLASMODIUM FALCIPARUM 17-33: YLEKQQSAWPFLKPVSL (#12)

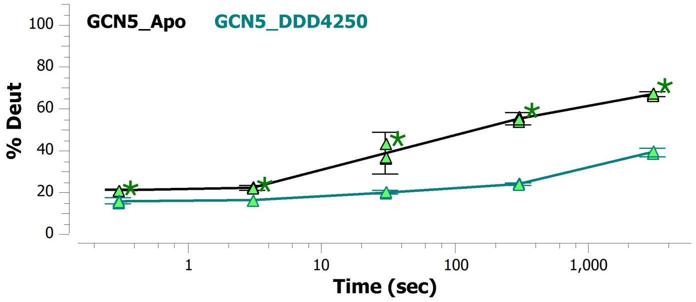

GCN5_PLASMODIUM FALCIPARUM 18-25: LEKQQSAW (#13)

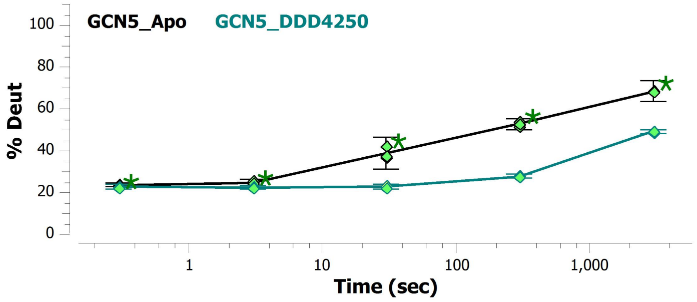

GCN5_PLASMODIUM FALCIPARUM 18-28: LEKQQSAWPFL (#14)

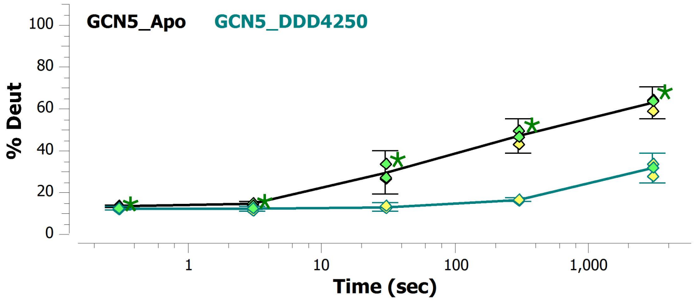

GCN5_PLASMODIUM FALCIPARUM 18-33: LEKQQSAWPFLKPVSL (#15)

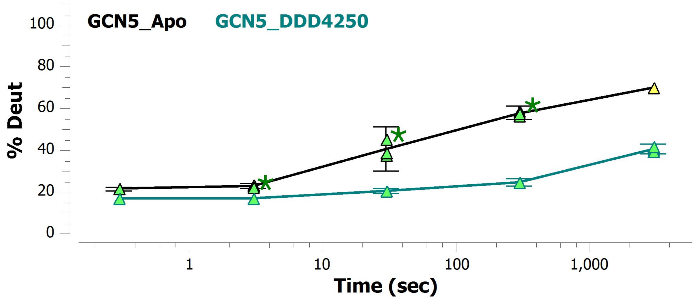

GCN5_PLASMODIUM FALCIPARUM 19-28: EKQQSAWPFL (#16)

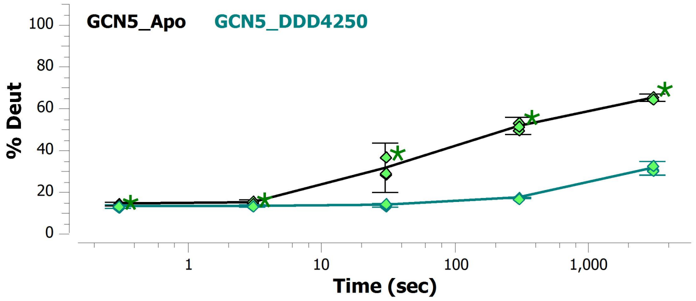

GCN5_PLASMODIUM FALCIPARUM 19-33: EKQQSAWPFLKPVSL (#17)

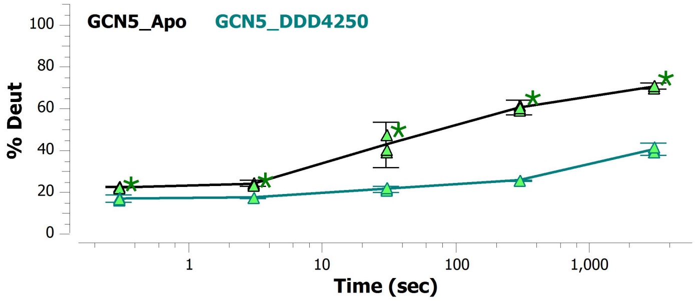

GCN5_PLASMODIUM FALCIPARUM 20-28: KQQSAWPFL (#18)

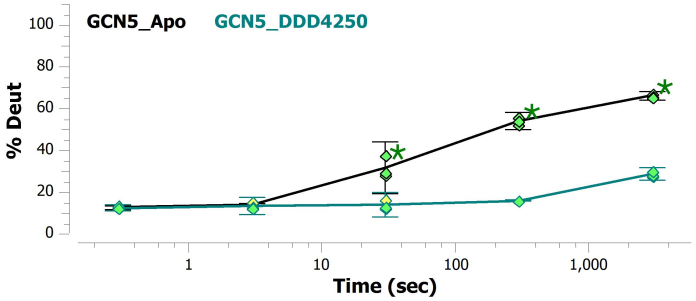

GCN5_PLASMODIUM FALCIPARUM 20-33: KQQSAWPFLKPVSL (#19)

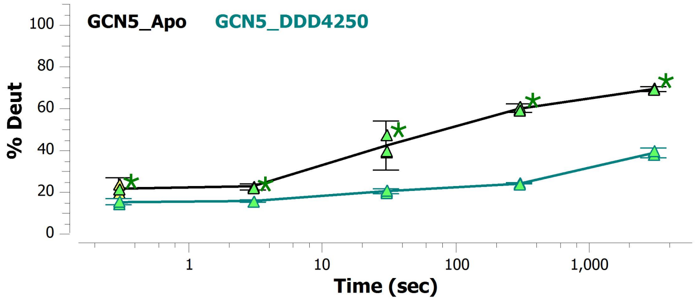

GCN5_PLASMODIUM FALCIPARUM 20-39: KQQSAWPFLKPVSLSEAPDY (#20)

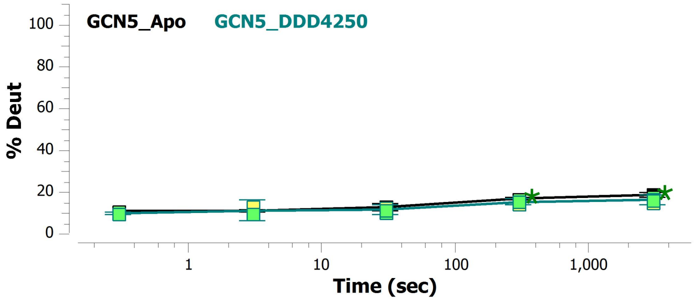

GCN5_PLASMODIUM FALCIPARUM 26-33: PFLKPVSL (#21)

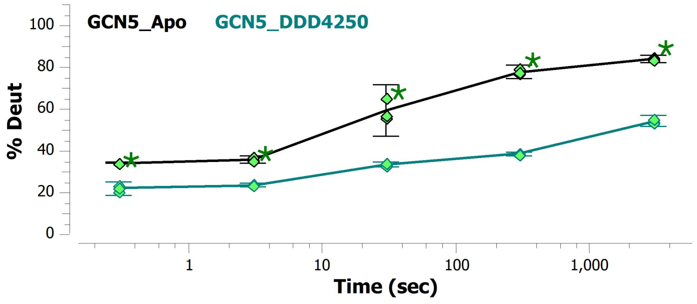

GCN5_PLASMODIUM FALCIPARUM 34-39: SEAPDY (#22)

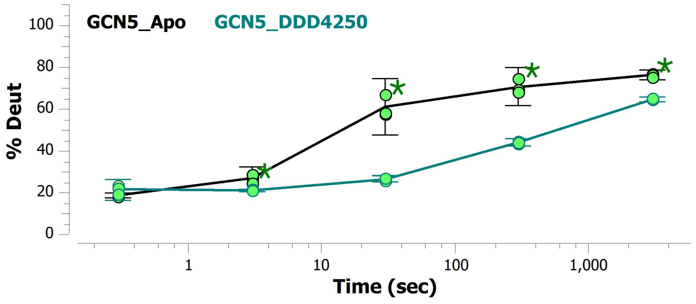

GCN5_PLASMODIUM FALCIPARUM 40-48: YDIIKEPTD (#23)

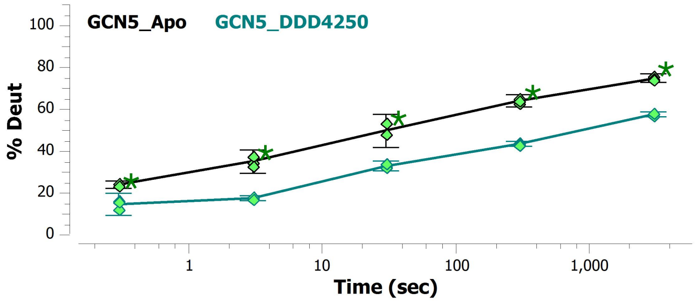

GCN5_PLASMODIUM FALCIPARUM 40-49: YDIIKEPTDI (#24)

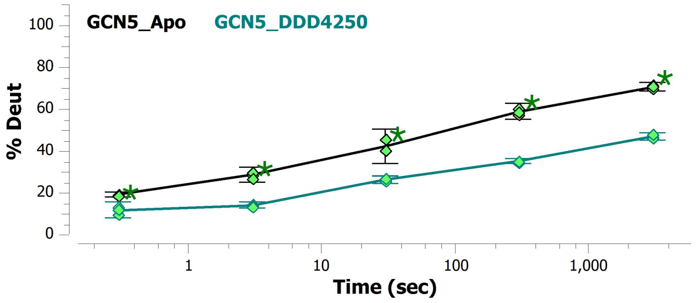

GCN5_PLASMODIUM FALCIPARUM 40-50: YDIIKEPTDIL (#25)

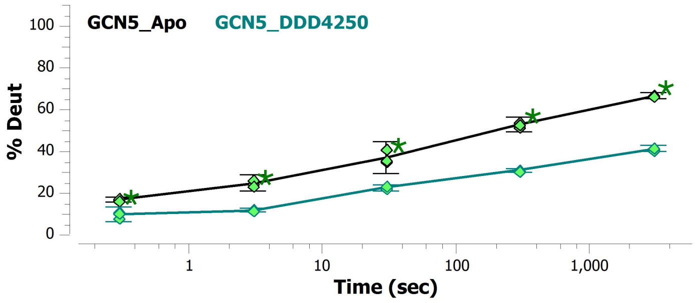

GCN5_PLASMODIUM FALCIPARUM 40-51: YDIIKEPTDILT (#26)

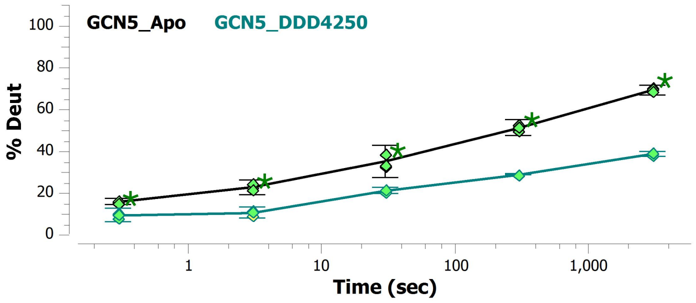

GCN5_PLASMODIUM FALCIPARUM 41-50: DIIKEPTDIL (#27)

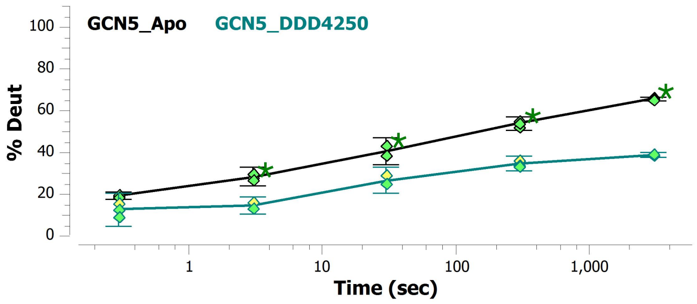

GCN5_PLASMODIUM FALCIPARUM 41-51: DIIKEPTDILT (#28)

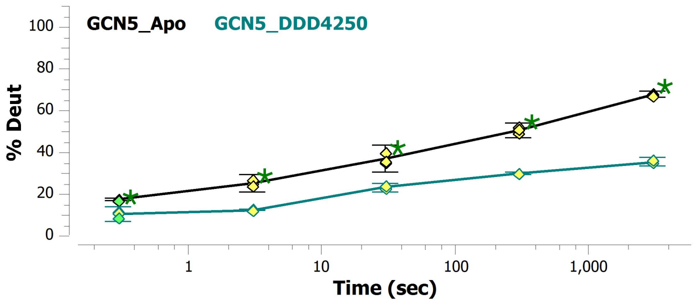

GCN5_PLASMODIUM FALCIPARUM 42-48: IIKEPTD (#29)

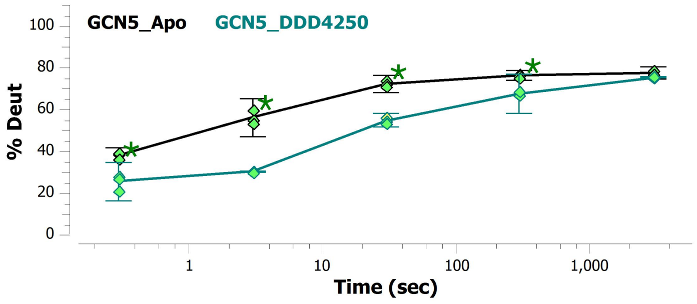

GCN5_PLASMODIUM FALCIPARUM 42-49: IIKEPTDI (#30)

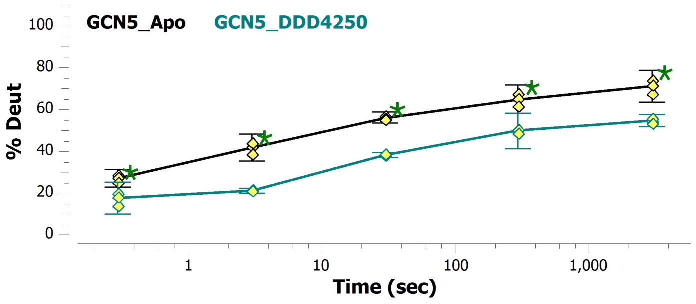

GCN5_PLASMODIUM FALCIPARUM 42-50: IIKEPTDIL (#31)

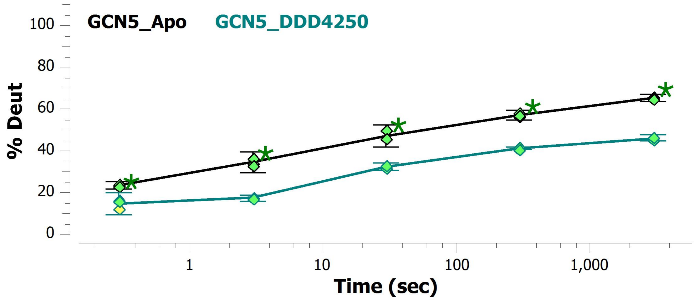

GCN5_PLASMODIUM FALCIPARUM 42-51: IIKEPTDILT (#32)

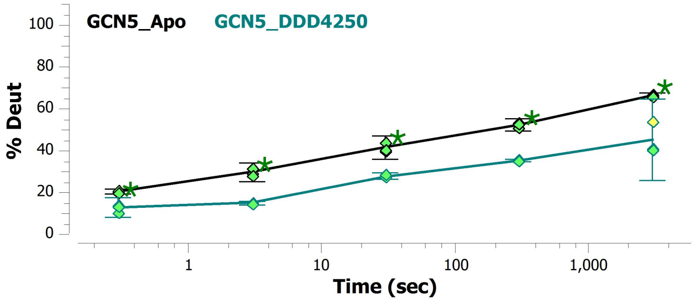

GCN5_PLASMODIUM FALCIPARUM 42-52: IIKEPTDILTM (#33)

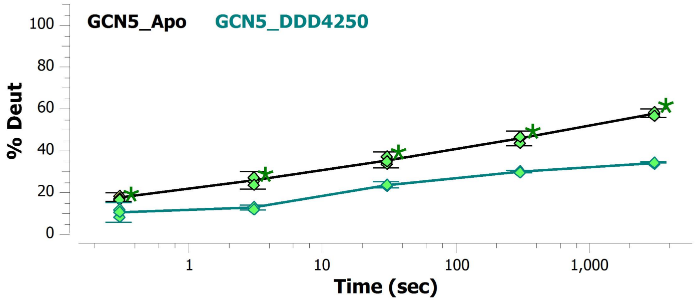

GCN5_PLASMODIUM FALCIPARUM 45-53: EPTDILTMR (#34)

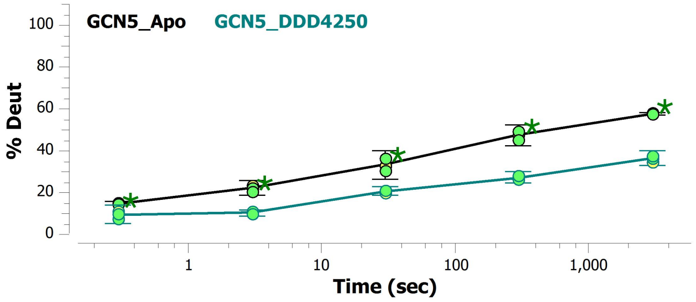

GCN5_PLASMODIUM FALCIPARUM 47-64: TDILTMRRKARHGDYKTK (#35)

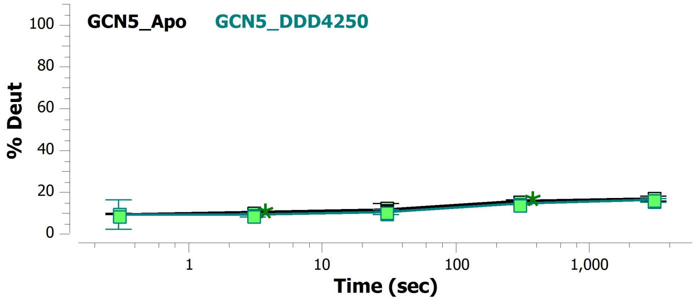

GCN5_PLASMODIUM FALCIPARUM 49-67: ILTMRRKARHGDYKTKEDF (#36)

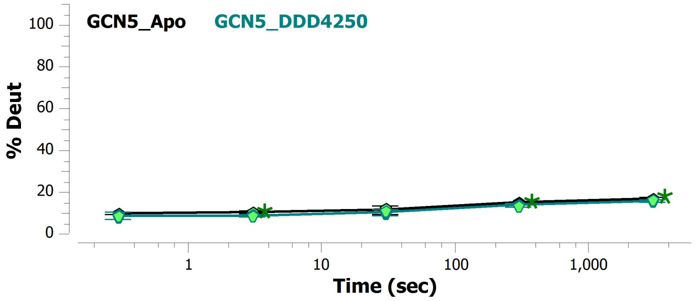

GCN5_PLASMODIUM FALCIPARUM 49-70: ILTMRRKARHGDYKTKEDFGIE (#37)

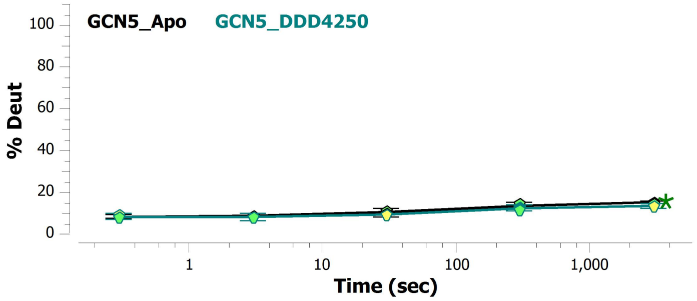

GCN5_PLASMODIUM FALCIPARUM 51-66: TMRRKARHGDYKTKED (#38)

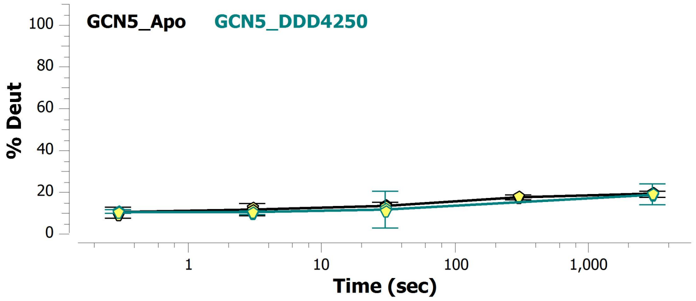

GCN5_PLASMODIUM FALCIPARUM 51-67: TMRRKARHGDYKTKEDF (#39)

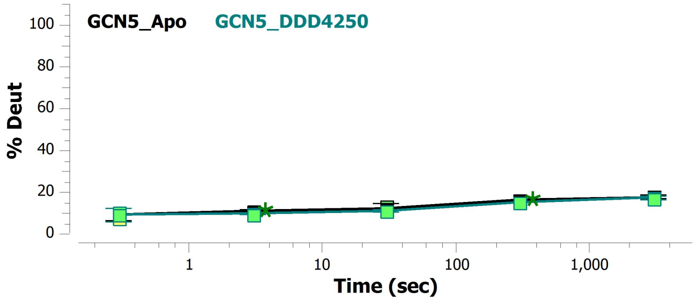

GCN5_PLASMODIUM FALCIPARUM 51-68: TMRRKARHGDYKTKEDFG (#40)

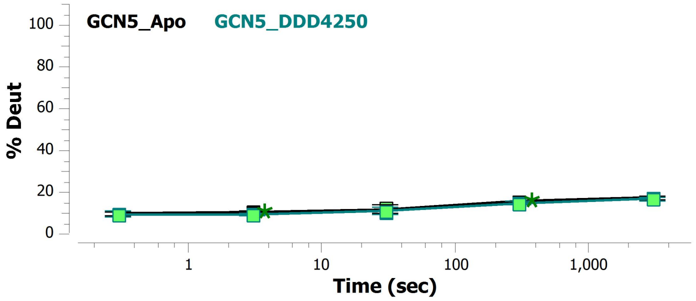

GCN5_PLASMODIUM FALCIPARUM 51-69:
TMRRKARHGDYKTKEDFGI (#41)

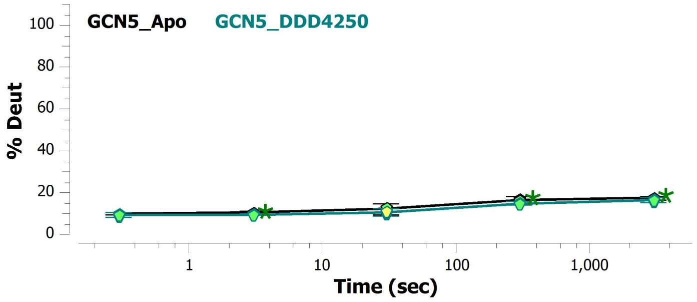

GCN5_PLASMODIUM FALCIPARUM 51-70:
TMRRKARHGDYKTKEDFGIE (#42)

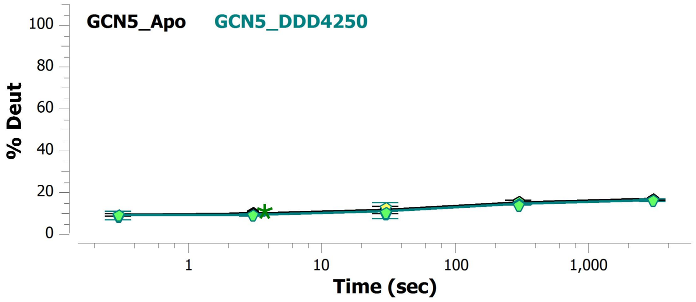

GCN5_PLASMODIUM FALCIPARUM 51-71:
TMRRKARHGDYKTKEDFGIEL (#43)

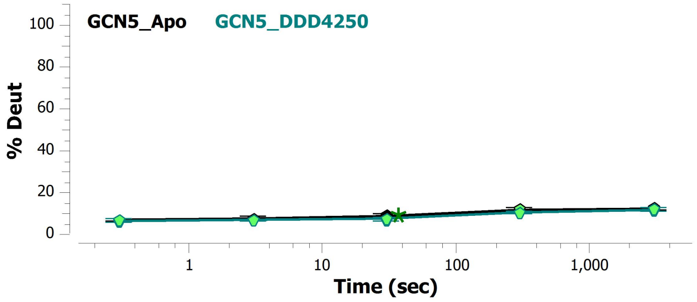

GCN5_PLASMODIUM FALCIPARUM 52-67:
MRRKARHGDYKTKEDF (#44)

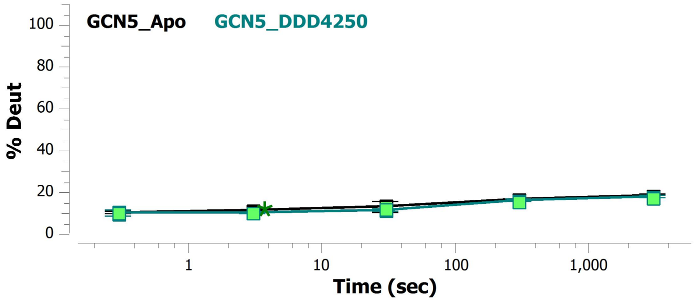

GCN5_PLASMODIUM FALCIPARUM 52-68:
MRRKARHGDYKTKEDFG (#45)

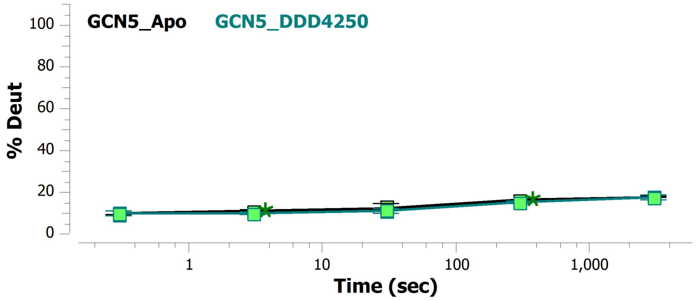

GCN5_PLASMODIUM FALCIPARUM 52-70:
MRRKARHGDYKTKEDFGIE (#46)

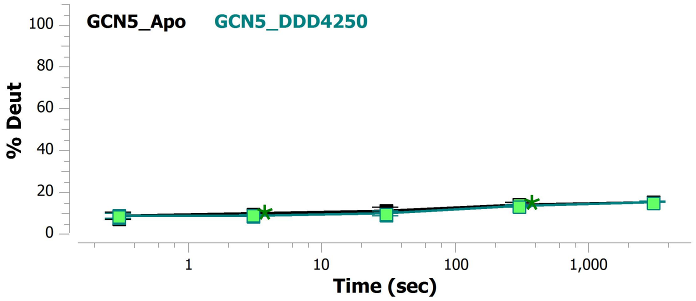

GCN5_PLASMODIUM FALCIPARUM 52-71:
MRRKARHGDYKTKEDFGIEL (#47)

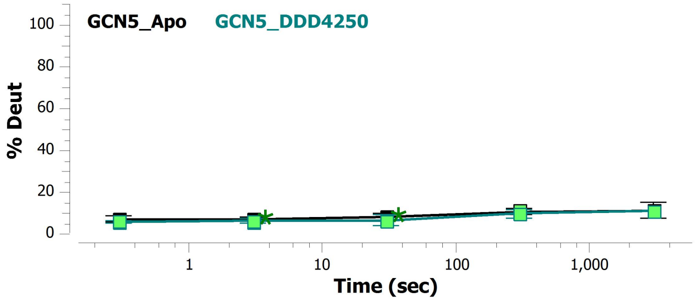

GCN5_PLASMODIUM FALCIPARUM 53-68:
RRKARHGDYKTKEDFG (#48)

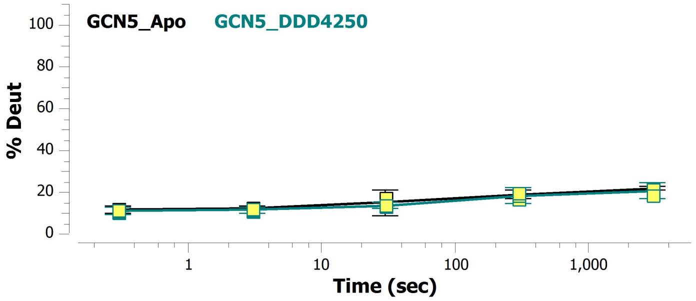

GCN5_PLASMODIUM FALCIPARUM 53-70:
RRKARHGDYKTKEDFGIE (#49)

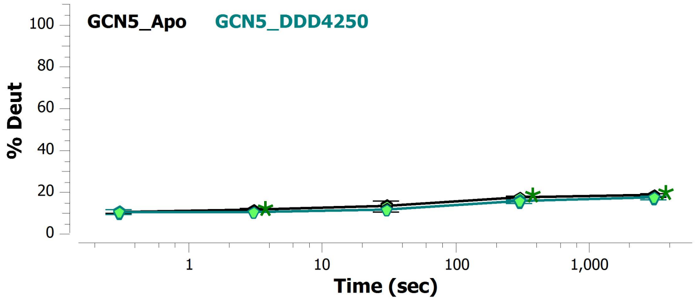

GCN5_PLASMODIUM FALCIPARUM 59-75:
GDYKTKEDFGIELKRMF (#50)

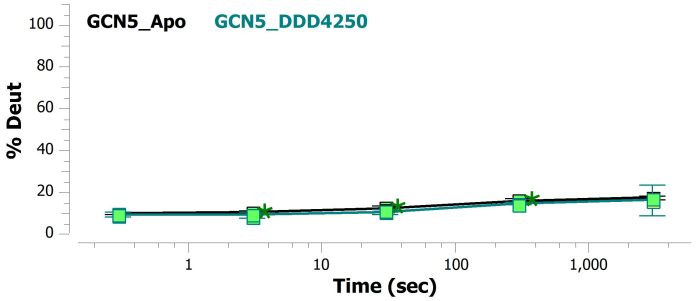

GCN5_PLASMODIUM FALCIPARUM 71-78: LKRMFDNC (#51)

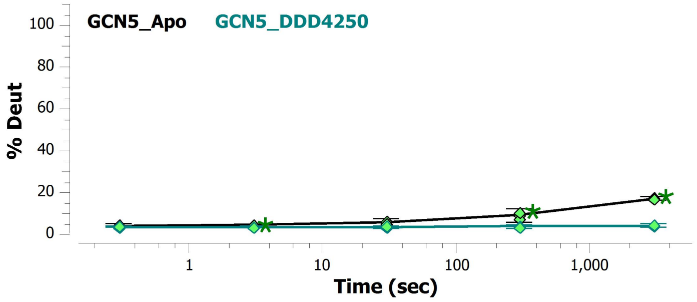

GCN5_PLASMODIUM FALCIPARUM 71-80: LKRMFDNCRL (#52)

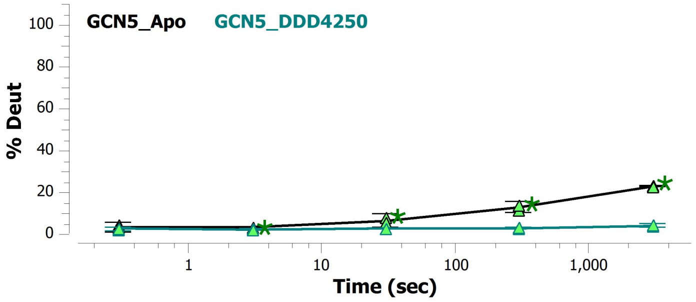

GCN5_PLASMODIUM FALCIPARUM 72-78: KRMFDNC (#53)

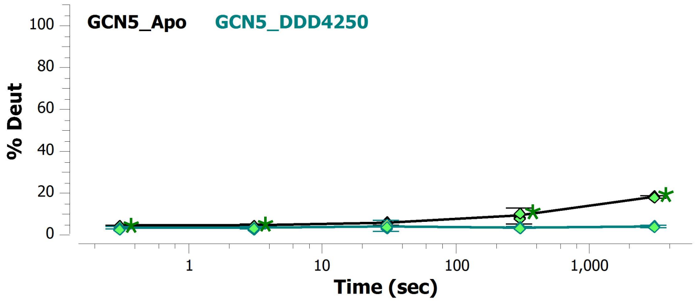

GCN5_PLASMODIUM FALCIPARUM 72-79: KRMFDNCR (#54)

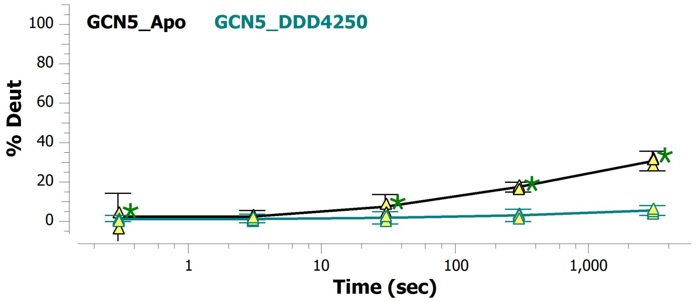

GCN5_PLASMODIUM FALCIPARUM 72-80: KRMFDNCRL (#55)

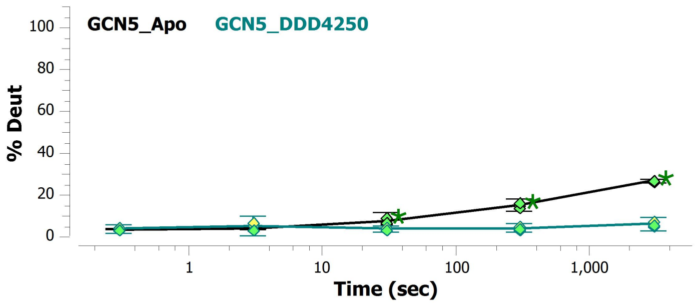

GCN5_PLASMODIUM FALCIPARUM 73-80: RMFDNCRL (#56)

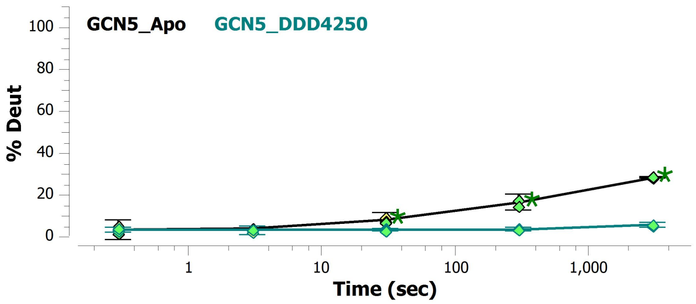

GCN5_PLASMODIUM FALCIPARUM 79-88: RLYNAPTTIY (#57)

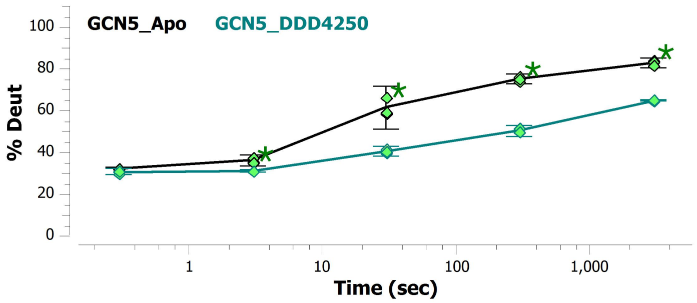

GCN5_PLASMODIUM FALCIPARUM 79-95: RLYNAPTTIYFKYANEL (#58)

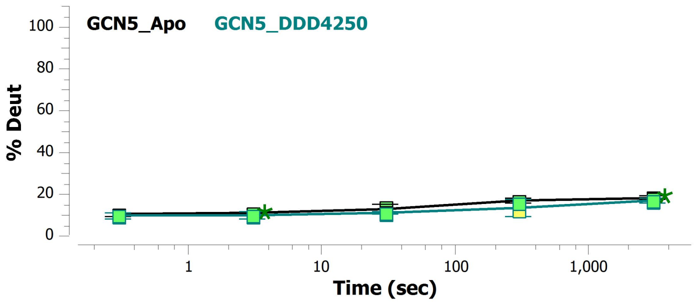

GCN5_PLASMODIUM FALCIPARUM 80-98: LYNAPTTIYFKYANELQTL (#59)

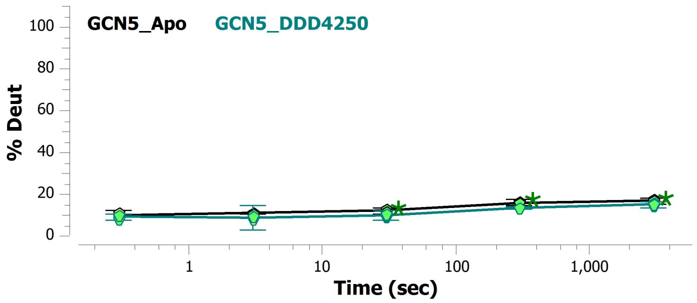

GCN5_PLASMODIUM FALCIPARUM 81-89: YNAPTTIYF (#60)

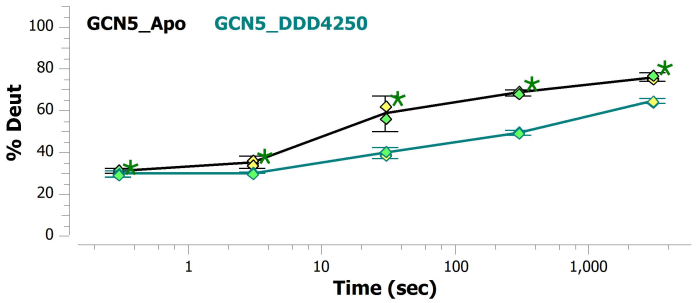

GCN5_PLASMODIUM FALCIPARUM 81-98: YNAPTTIYFKYANELQTL (#61)

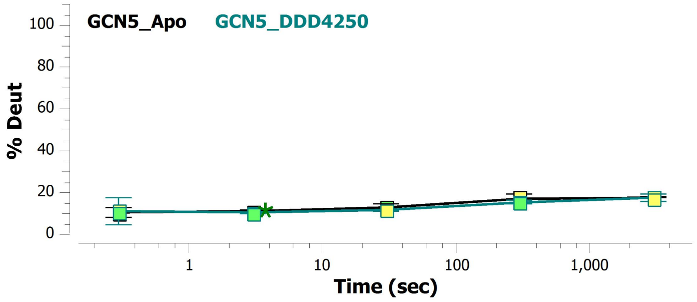

GCN5_PLASMODIUM FALCIPARUM 81-99: YNAPTTIYFKYANELQTLI (#62)

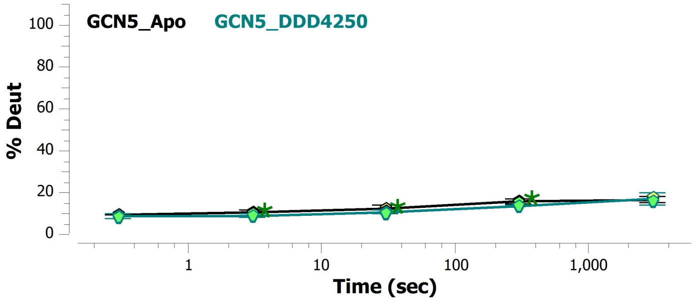

GCN5_PLASMODIUM FALCIPARUM 83-98: APTTIYFKYANELQTL (#63)

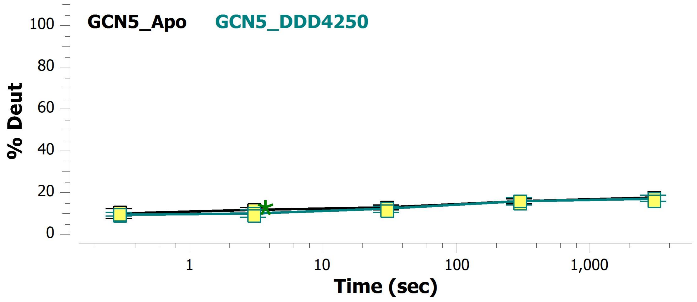

GCN5_PLASMODIUM FALCIPARUM 86-103: TIYFKYANELQTLIWPKY (#64)

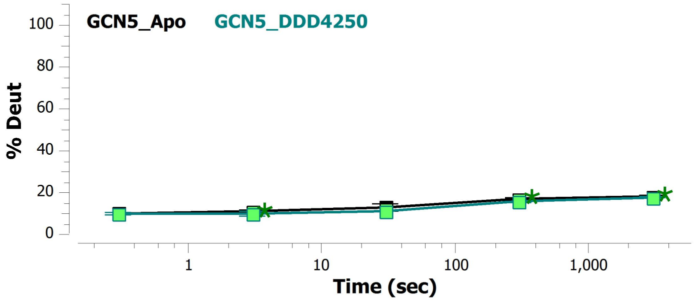

GCN5_PLASMODIUM FALCIPARUM 87-94: IYFKYANE (#65)

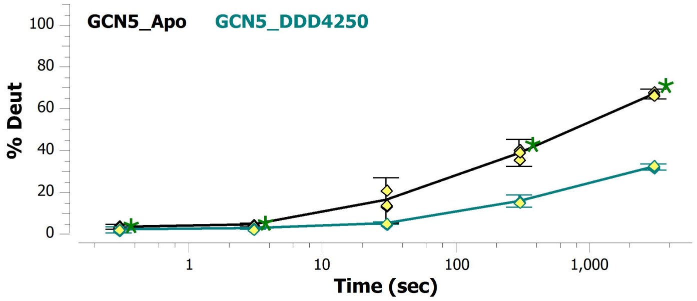

GCN5_PLASMODIUM FALCIPARUM 87-104: IYFKYANELQTLIWPKYE (#66)

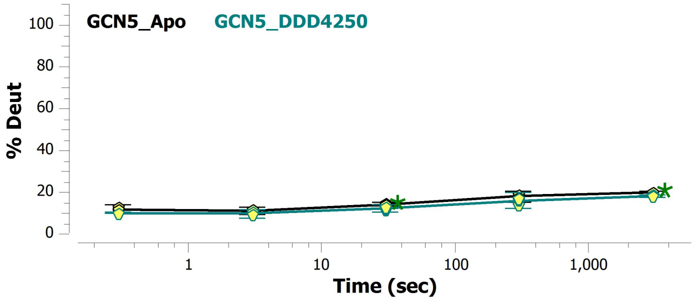

GCN5_PLASMODIUM FALCIPARUM 88-94: YFKYANE (#67)

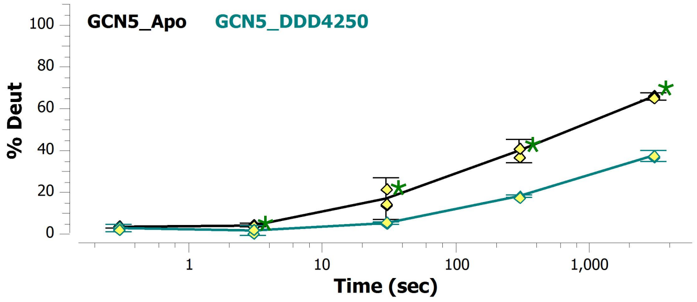

GCN5_PLASMODIUM FALCIPARUM 89-95: FKYANEL (#68)

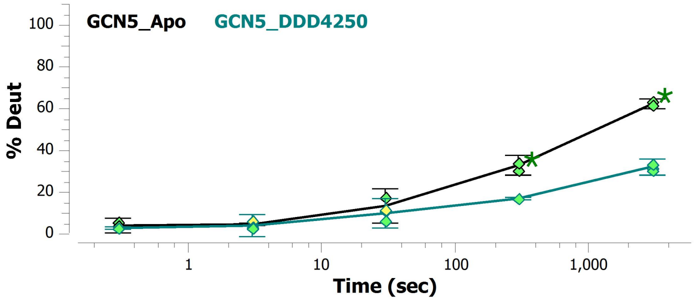

GCN5_PLASMODIUM FALCIPARUM 96-103: QTLIWPKY (#69)

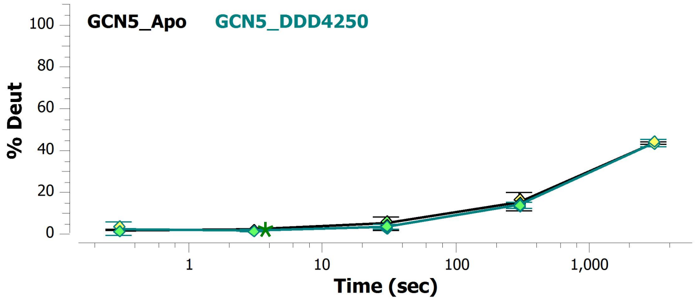

GCN5_PLASMODIUM FALCIPARUM 96-106: QTLIWPKYEAI (#70)

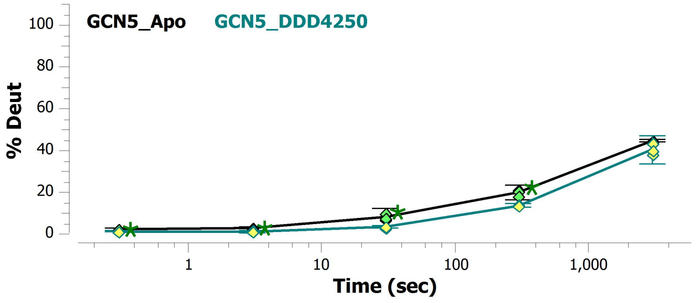

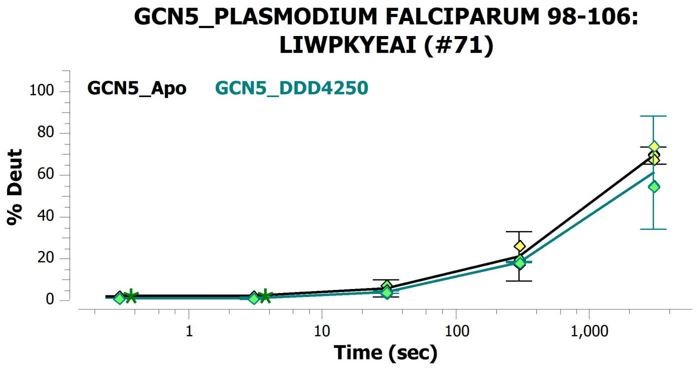

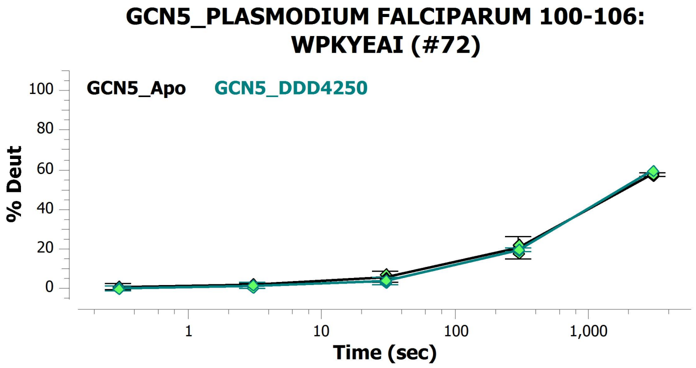

Figure S4: *Deuteration uptake graphs of PfGCN5-BRD with 120 µM ‘4250. Each point is the average of three independent exchange reactions. Error bars are standard deviation. * = passes t-test (as performed in HDExaminer Software).*
